# CRISPR-based screening of small RNA modulators of bile susceptibility in *Bacteroides thetaiotaomicron*

**DOI:** 10.1073/pnas.2311323121

**Published:** 2024-01-31

**Authors:** Gianluca Prezza, Chunyu Liao, Sarah Reichardt, Chase L. Beisel, Alexander J. Westermann

**Affiliations:** ^a^Helmholtz Institute for RNA-based Infection Research, Helmholtz Centre for Infection Research, Würzburg D-97080, Germany; ^b^Medical Faculty, University of Würzburg, Würzburg D-97080, Germany; ^c^Institute of Molecular Infection Biology, University of Würzburg, Würzburg D-97080, Germany; ^d^Department of Microbiology, Biocentre, University of Würzburg, Würzburg D-97074, Germany

**Keywords:** Bacteroides, CRISPRi, sRNA, thetaiotaomicron, microbiota

## Abstract

Microbiota-centric interventions are limited by our incomplete understanding of the gene functions of many of its constituent species. This applies in particular to small RNAs (sRNAs), which are emerging as important regulators in microbiota species yet tend to be missed by traditional functional genomics approaches. Here, we establish CRISPR interference (CRISPRi) in the abundant microbiota member *Bacteroides thetaiotaomicron* for genome-wide sRNA screens. By assessing the abundance of different protospacer-adjacent motifs, we identify the *Prevotella bryantii* B14 Cas12a as a suitable nuclease for CRISPR screens in these bacteria and generate an inducible Cas12a expression system. Using a luciferase reporter strain, we infer guide design rules and use this knowledge to assemble a computational pipeline for automated gRNA design. By subjecting the resulting guide library to a phenotypic screen, we uncover the sRNA BatR to increase susceptibility to bile salts through the regulation of genes involved in *Bacteroides* cell surface structure. Our study lays the groundwork for unlocking the genetic potential of these major human gut mutualists and, more generally, for identifying hidden functions of bacterial sRNAs.

## Significance

Bacterial genomes universally code for small noncoding RNAs (sRNAs) that posttranscriptionally regulate mRNA targets through sequence complementarity. While the importance of sRNA genes has been recognized in both model and noncanonical species, tools for their global functional characterization have been lacking. Here, we employ CRISPR interference (CRISPRi) to generate a targeted knockdown library of the intergenic sRNA repertoire of *Bacteroides thetaiotaomicron* and use it to identify sRNAs regulating *Bacteroides* physiology under bile stress. This work illustrates the use of CRISPRi for the functional characterization of sRNAs and provides a foundation for the replication of this approach in other bacterial species.

The human intestinal microbiota is dominated by two bacterial phyla, the Firmicutes and the Bacteroidetes (1). Among the latter, obligate anaerobic gut *Bacteroides* are of paramount importance for human health and disease (2). To exploit these bacteria for the benefit of the host, we first need to decipher the functions of their genes. While we still have an incomplete understanding of the function of many protein-coding genes expressed by *Bacteroides* spp., there is barely any knowledge as to the function of noncoding genes, even in emerging microbiological model organisms such as *Bacteroides thetaiotaomicron* (3, 4).

The best-understood and most prevalent class of noncoding RNA regulators in the bacterial kingdom are the small RNAs (sRNAs). These ~50 to 250 nt-long RNA molecules typically regulate target gene expression through base-pair interactions with complementary stretches within target mRNAs and control a wide range of cellular processes (5–7). For example, enterobacterial model species often deploy sRNAs to rapidly adapt their global gene expression to numerous intrinsic or environmental stress conditions (8). By contrast, only a handful of sRNAs have been characterized in *Bacteroides* spp. (9, 10). While functional genomics proved successful to link *Bacteroides* coding genes with phenotypes (11, 12), conventional approaches—such as transposon insertion-based perturbations (13, 14)—inactivate genes near-randomly. Consequently, in these approaches, the likelihood of a given gene being hit and inactivated is directly proportional to its length. This renders such screens less practical for studying short genes, including genes encoding small proteins (15) or sRNAs. For example, of the currently annotated 135 intergenic *B*. *thetaiotaomicron* sRNAs, 54 were missed in a recent dense (315,668 unique mutants; on average, one insertion every ~20 nucleotides) transposon insertion sequencing (TIS) screen (10, 12). By extrapolation, it follows that roughly 2.5 times more mutants would be minimally required for a transposon library to hit all annotated sRNAs in this organism. Even if such a library would be available, parallel screening of that many mutants would heavily increase the already large bottleneck effects that such approaches suffer from (14).

Instead, CRISPR interference (CRISPRi)—wherein specific guide RNAs (gRNAs) recruit catalytically inactive CRISPR-associated (Cas) nuclease molecules to interfere with target gene transcription (16)—lends itself for the knockdown of short genes. The minimal requirement for CRISPRi-based target gene suppression is the presence of a protospacer adjacent motif (PAM), which is usually composed of only a few nucleotides. The resulting targeting scope thus entails the entire genome, with the added advantage of tailored knockdowns of predefined gene sets. Recently, using a catalytically dead *Streptococcus pyogenes* Cas9 (dCas9) and gRNA expression vectors, a proof of principle was provided for CRISPRi in *Bacteroides* spp. (17). However, as of now the technology has not been exploited for functional screening in these important gut mutualists. More generally, in spite of ongoing attempts (18, 19), to our knowledge, CRISPRi has not previously been used for a systematic assessment of sRNA-associated phenotypes in any bacterial species.

Here, we deploy CRISPRi to knock down the full complement of known intergenic sRNAs of *B*. *thetaiotaomicron* type strain VPI-5482 for fitness screening. Of six Cas nucleases considered for CRISPRi, *Prevotella bryantii* B14 Cas12a (Pb2Cas12) recognizes a PAM that is the most represented within the 135 annotated intergenic *B*. *thetaiotaomicron* sRNAs. Combining an inducible dPb2Cas12a expression system with a luciferase reporter strain, we infer guide design rules for CRISPRi in *B*. *thetaiotaomicron* and employ this knowledge to generate a computational pipeline for automated library design. We use the resulting guide library to identify sRNAs whose repression affects *Bacteroides* resilience to bile stress. Through the resulting screen, we identify the previously uncharacterized sRNA BatR, which regulates genes involved in *Bacteroides* cell surface biosynthesis/assembly and confers enhanced susceptibility to bile salts. Altogether, the present guide library bears potential to systematically uncover phenotypes for *Bacteroides* sRNAs under a variety of experimental conditions. More generally, our work lays the ground for targeted gene knockdown in these abundant human microbiota members.

## Results

### CRISPR Nuclease Selection and Induced Expression.

We recently annotated a total of 366 noncoding genes in the genome of *B*. *thetaiotaomicron* type strain VPI-5482 (10). This list includes 156 *cis*-encoded antisense RNAs, 28 5′ UTR-derived and 24 3′ UTR-derived sRNAs. Targeted disruption of these noncoding RNA types may potentially affect the expression of the correspondingly overlapping protein-coding genes, thereby complicating the interpretation of possible phenotypes. Instead, we here focused on the 135 *B*. *thetaiotaomicron* sRNAs that do not overlap any other annotated gene, to which we refer to as “intergenic” sRNAs (10).

**Fig. 1.**
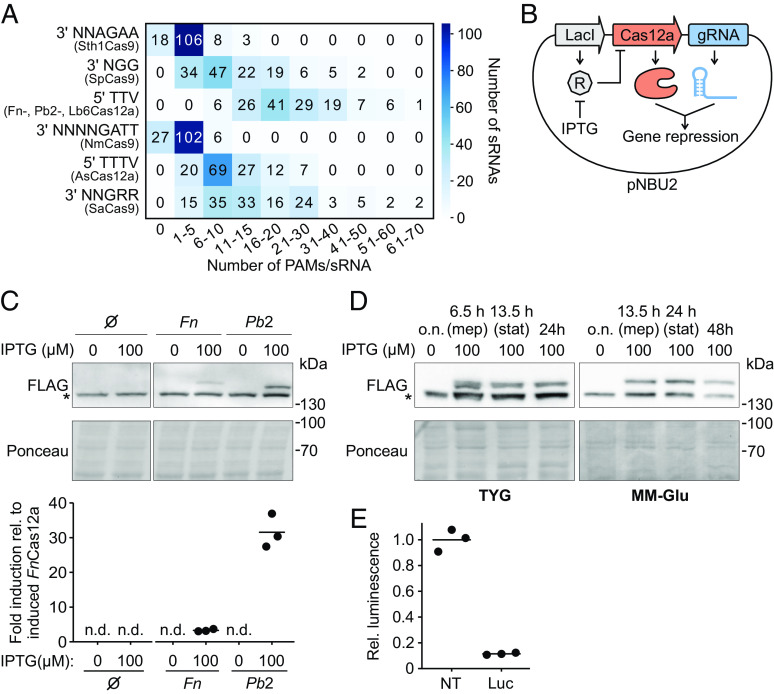
Nuclease selection for CRISPRi in *Bacteroides*. (*A*) Frequency of PAM occurrences within the intergenic sRNAs of *B*. *thetaiotaomicron*. Color intensity is directly proportional to the number of sRNAs that contain the PAMs indicated on the *y* axis. Examples of Cas nucleases recognizing each PAM are listed below the PAM sequence. (*B*) Scheme of the construct for inducible and constitutive expression of Cas12a and gRNAs in *B*. *thetaiotaomicron*, respectively. The *lacI* gene produces a repressor (R) that blocks Cas12a expression. IPTG added to the culture media sequesters the repressor, allowing Cas12a transcription. A downstream gRNA is expressed from a constitutive promoter. Cas12a and the gRNA form an RNP complex that represses target gene transcription. (*C*) *Top*: Expression of FLAG-tagged FnCas12a and Pb2Cas12a in *B*. *thetaiotaomicron* at mid-exponential phase (OD_600_ = 2, ~6.5 h after IPTG induction). A sample harboring an empty construct (∅) is included as a control. The asterisk next to the blot marks an unspecific band. *Bottom*: Quantification of Cas12a band intensities (relative to induced FnCas12a). Horizontal lines represent the mean values of three biological replicates. n.d., not detected. (*D*) Detection of Pb2Cas12a in *B*. *thetaiotaomicron* in rich (*Left*, TYG) or minimal medium with glucose as sole carbon source (*Right*, MM-Glu) after initial IPTG induction. The immunoblot is representative of three biological replicates and the asterisk marks an unspecific band. (*E*) Luciferase activity after expression of a nontargeting control gRNA (NT) or a luciferase-targeting gRNA (Luc). Values are relative to the NT gRNA. Horizontal lines represent the means of three biological replicates.

The low GC content of *B*. *thetaiotaomicron* intergenic sRNAs [35% (20)] likely results in an underrepresentation of the 5′-NGG-3′ PAM recognized by the most frequently used Cas9 nuclease from *Streptococcus pyogenes* (SpCas9). In order to maximize the number of potential targeting sites, we screened the intergenic sRNAs for the presence of PAMs of additional, characterized CRISPR effector nucleases, including 5′-NNAGAA-3′ [recognized by Sth1Cas9 (21)], 5′-TTV-3′ [FnCas12a, Pb2Cas12a, and Lb6Cas12a (22)], 5′-NNNNGATT-3′ [NmCas9 (23)], 5′-TTTV-3′ [AsCas12a (24)], and 5′-NGRR-3′ [SaCas9 (25)] (Fig. 1*A*). The SpCas9 PAM had a moderate abundancy, with most sRNAs having a limited number of Cas9-PAMs. In contrast, the Cas12a-specific PAM 5′-TTV-3′ was the most frequent and present at least 6 times in each sRNA. Overall, this PAM remained the top hit when expanding the search space to the entire *B*. *thetaiotaomicron* genome sequence (*SI Appendix*, Fig. S1*A*), proposing Cas12a as a suitable nuclease for genome-wide *Bacteroides* screens in general terms.

Using an *E*. *coli* cell-free transcription-translation (TXTL) system (22, 26) as an initial screening tool, we tested the cleavage efficiency of three phylogenetically distinct Cas12a orthologs that recognize the same 5′-TTV-3′ PAM (*Lachnospiraceae bacterium* COE1, Lb6Cas12a; *Prevotella bryantii* B14, Pb2Cas12a; *Francisella novicida* U112, FnCas12a) (22) (*SI Appendix*, Fig. S1*B*). Of these orthologs, only Pb2Cas12a and FnCas12a yielded substantial cleavage of a targeted GFP reporter plasmid (*SI Appendix*, Fig. S1*C*). Next, we cloned Pb2Cas12a and FnCas12a into a pNBU2-based *Bacteroides* integration vector that allows expression of the nuclease from an IPTG-inducible promoter, while constitutively expressing a luciferase reporter gene and its corresponding gRNA (17) (Fig. 1*B*). After IPTG induction, both nucleases were detectable via western blotting, although Pb2Cas12a accumulated to a considerably higher level (Fig. 1*C*). We therefore selected this ortholog for CRISPRi. Upon induction with IPTG at a concentration of 250 µM—sufficient to achieve maximum expression of the nuclease (*SI Appendix*, Fig. S1*D*)—we detected the Pb2Cas12a protein at near-constant levels for at least 24 h of growth in rich medium and for 48 h in defined minimal medium with glucose (Fig. 1*D*).

CRISPRi uses a catalytically inactive nuclease variant to sterically interfere with RNA polymerase-mediated transcription without cleaving the targeted DNA. Cas12a proteins have a single RuvC-like DNA cleavage domain (24), which can be inactivated with a single amino acid exchange (D917A in FnCas12a), thereby abrogating the protein’s catalytic activity (24). In analogy to the D917A FnCas12a variant, we introduced the D875A mutation into Pb2Cas12a (giving rise to “deactivated” Pb2Cas12a [dPb2Cas12a]). Recruiting dPb2Cas12a to the promoter of the luciferase reporter construct resulted in a luminescence signal reduction of ~10-fold (Fig. 1*E*). Correlating the dPb2Cas12a protein abundance with knockdown efficiency revealed that maximal target repression is reached with IPTG concentrations of ≥25 µM (*SI Appendix*, Fig. S1*E*). Retrospectively, this further supports our choice against FnCas12a since its low expression (Fig. 1*C*) would likely impede CRISPRi efficiency. Together, these advances position dPb2Cas12a as a preferred nuclease for CRISPRi in *Bacteroides* for functional screening.

### Inferring gRNA Design Rules and Construction of CRISPR Arrays.

**Fig. 2.**
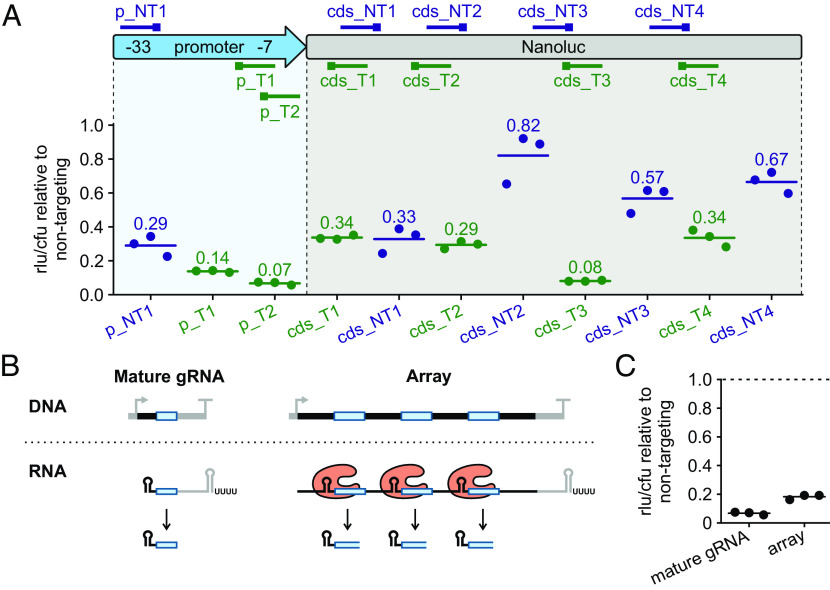
Targeting rules and array design for dPb2Cas12a*.* (*A*) Knockdown of luciferase by dPb2Cas12a with different gRNAs. *Top*: Scheme of the luciferase gene and promoter with the relative position of the targeted regions. The position of the PAM is marked with a square. gRNAs shown in blue and green target the nontemplate and template strands, respectively. The –33 and –7 promoter regions are highlighted. *Bottom*: Detected luciferase activity after expression of each gRNA. Values are relative to the levels observed in a strain encoding a nontargeting gRNA. Horizontal lines mark the means of three biological replicates. (*B*) Depiction of the expression of a single gRNA (*Left*) or three gRNAs from an array (*Right*). In the latter case, the single array transcript is recognized and processed by Cas12a into the three independent gRNA units. (*C*) Knockdown of luciferase by dPb2Cas12a with the same spacer expressed as a mature gRNA or surrounded by *Francisella* repeats in a CRISPR array-like transcript. The data of the mature gRNA are the same as shown in Fig. 2*A* for “p_T2”. Values are relative to levels observed in a strain with a nontargeting spacer (dashed line), encoded either as gRNA or as an array with *Francisella* repeats (n = 3).

As part of CRISPRi, the efficiency of gene knockdown is often dependent on the identity of the DNA strand that is targeted, with this specificity varying among Cas nucleases. Previous bacterial CRISPRi studies based on different Cas12a orthologs (FnCas12a, LbCas12a, and AsCas12a) revealed that efficient knockdown can be achieved by targeting either strand within the promoter region (27–29). In contrast, when targeting within transcribed regions, knockdown was only effective when using gRNAs annealing to the template strand. To evaluate whether these findings can be extrapolated to the use of dPb2Cas12a in *B*. *thetaiotaomicron*, we designed eleven gRNAs complementary to either DNA strand within the promoter or coding region of the luciferase gene in our reporter strain (Fig. 2*A*). Compared to a nontargeting control gRNA, the gRNAs targeting either DNA strand in the promoter reduced the luciferase signal intensity by ~3- to 10-fold. Within the coding region, gRNAs annealing to the template strand were more effective than those against the nontemplate strand. The only exception was gRNA “cds_NT1,” which anneals to the nontemplate strand in the 5′ portion of the coding region but still resulted in a threefold repression. This may be due to the proximity to the promoter region and/or the presence of the extended PAM “TTTV,” which is slightly preferred over “VTTV” in *E*. *coli* (22). Based on these results, we concluded that in our system Cas12a-mediated transcriptional interference shows similar strand preferences than previously reported for other bacterial taxa and Cas12a orthologs.

One advantage of Cas12a over other Cas nucleases is its ability to process CRISPR arrays without the requirement of further accessory factors (30). Therefore, as an alternative to coexpressing mature gRNAs individually (Fig. 2 *B*, *Left*), expression of a CRISPR array would eventually result in the accumulation of multiple processed gRNAs starting from a single, compact transcript (Fig. 2 *B*, *Right*) (24). We explored to what extent this property of Cas12a can be exploited for simultaneous expression in *B*. *thetaiotaomicron* of multiple gRNAs derived from a single primary transcript. To this end, we designed an array composed of a spacer targeting the luciferase promoter (“p_T2” in Fig. 2*A*) surrounded by either *Francisella* or *Prevotella* Cas12a repeats. As revealed by northern blotting, the *Francisella* array was efficiently processed into mature gRNAs upon dPb2Cas12a induction, whereas no processing was observed in case of the *Prevotella* array (*SI Appendix*, Fig. S2*A*), in spite of its predicted proper folding (*SI Appendix*, Fig. S2*B*). Accordingly, only the array containing *Francisella* repeats (Fig. 2*C*)—yet not the one with the *Prevotella* repeats (*SI Appendix*, Fig. S2*C*)—resulted in luciferase knockdown levels close to the ones achieved by delivering the mature gRNA. While the reason for the different processing efficiencies remains elusive, we note that our observations are based on only these two arrays and should not be generalized. Importantly, since dPb2Cas12a proved capable of efficient gRNA maturation from *Francisella* arrays in *B*. *thetaiotaomicron*, we relied on this setup for all follow-up experiments.

### Efficient dCas12a-mediated Inhibition of sRNA Transcription and Function.

**Fig. 3.**
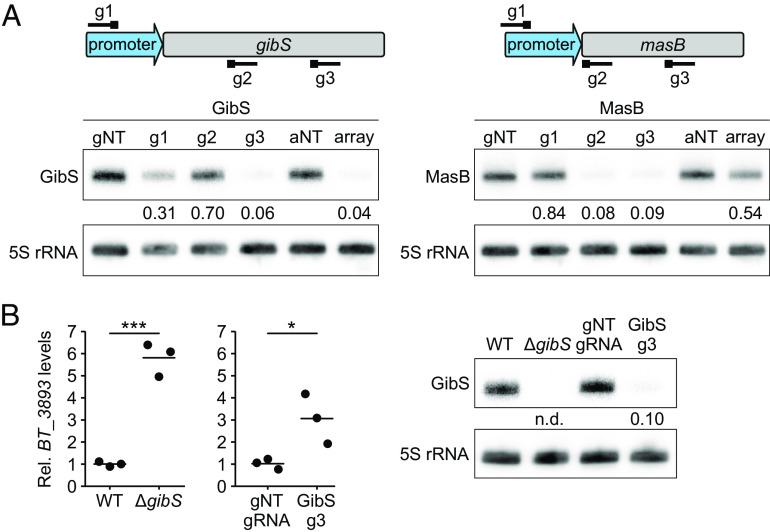
CRISPRi-mediated knockdown of selected *B*. *thetaiotaomicron* sRNAs. (*A*) Depletion of the sRNAs GibS (*Left*) and MasB (*Right*) with dPb2Cas12a as detected by northern blotting. For each sRNA, three gRNAs (“g1-3”) or an array (“array”) were used, alongside a nontargeting gRNA (“gNT”) or nontargeting array (“aNT”). The relative annealing position of each spacer within the sRNA genes and promoters is depicted at the top. The blots shown are representative of three biological replicates and the mean value of each band intensity relative to the respective control is indicated below the blot. (*B*) Recapitulation of a *gibS* deletion-associated phenotype with CRISPRi. *Left*: qRT-PCR measurements of *BT_3893* mRNA levels relative to the 16S rRNA in Δ*tdk* (WT), Δ*tdk*Δ*gibS* (Δ*gibS*) strains or in bacteria harboring the CRISPRi vector with a nontargeting (gNT gRNA) or a GibS-targeting (GibS g3) gRNA. Values shown are relative to Δ*tdk* or to the nontargeting gRNA-expressing strain, respectively. Horizontal lines represent the mean values from three biological replicates. ***P-value < 0.001, *P-value < 0.05; unpaired *t* test. *Right*: GibS levels as measured by northern blotting in the same samples. The blot is representative of three biological replicates and the mean of each band intensity relative to the respective control is indicated below the blot. n.d.: not detected.

Building on the targeting rules inferred above, we designed gRNAs against two well-established *B*. *thetaiotaomicron* sRNAs, GibS (20) and MasB (10) (Fig. 3 *A*, *Top*). In each case, we designed three spacers annealing to different regions within the targeted sRNA gene or its promoter and expressed them as single gRNAs (g1–3′). When compared to a nontargeting control (“gNT”), most of the gRNAs led to efficient (i.e., at least threefold) reduction in the respective sRNA levels (Fig. 3 *A*, *Bottom*), yet two gRNAs (“GibS g2” and “MasB g1”) resulted in negligible repression. While the cause of inefficient knockdown for these guides remains unknown, we note that by including multiple constructs targeting the same sRNA in the final library pool, the chances of failing to repress expression of a targeted sRNA are low.

We next assembled the three guides targeting the same sRNA into a CRISPR array. In the case of the *gibS*-targeting array (Fig. 3 *A*, *Left*), the steady-state level of the sRNA was reduced by ~20-fold as compared to an array encoding a nontargeting guide (“arrayNT”). However, the *masB*-targeting array failed to strongly suppress transcription of this sRNA (Fig. 3 *A*, *Right*), despite the fact that two of the contained guides led to a >10-fold repression when expressed as single gRNAs (“g2, -3”). Prediction of the arrays’ secondary structure revealed that all repeats in the *gibS*-targeting construct correctly fold and form the terminal 3′ hairpin required for array processing (24) in at least one of the most probable (based on minimal free energy) structures. In contrast, in case of the *masB*-targeting array, several repeats did not form the hairpin in any of the alternative structures (*SI Appendix*, Fig. S3*A*). Northern blotting with probes specific for each spacer of both arrays supported this prediction: all three mature anti-*gibS* gRNAs readily accumulated, while only one of the three anti-*masB* gRNAs was detected, and so at a very low level (*SI Appendix*, Fig. S3*B*). We concluded that in line with previous observations (31), correct folding of the primary transcript must be taken into account when designing Cas12a arrays.

As a proof of concept for our CRISPRi-based sRNA knockdown approach in *B*. *thetaiotaomicron*, we tested whether expression of an anti-*gibS* gRNA would replicate molecular phenotypes previously established for a clean deletion mutant of this sRNA (20). Indeed, CRISPRi-mediated knockdown led to derepression of the confirmed GibS target *BT_3893* mRNA (Fig. 3 *B*, *Left*). We did not observe a similar relief in repression of another established GibS target, *BT_0771* (*SI Appendix*, Fig. S3*C*), in spite of reduced GibS levels (Fig. 3 *B*, *Right*). However, we hypothesize that *BT_0771* mRNA has a higher affinity to GibS than *BT_3893*, so residual traces of the sRNA that remain upon knockdown would be sufficient to repress *BT_0771* but not *BT_3893*. Collectively, these results show that sRNAs can be efficiently silenced in *B*. *thetaiotaomicron* via dPb2Cas12a-mediated CRISPRi, which can replicate phenotypes associated with clean sRNA deletions.

### Automated Design of gRNAs against the Full Suite of Intergenic *B. thetaiotaomicron* sRNAs.

Based on the inferred design rules, we created a custom Python script to design a guide library against the 135 intergenic sRNAs of *B*. *thetaiotaomicron* (*SI Appendix*, Fig. S4*A*). For each queried sRNA, the script designs three gRNAs that may anneal to either strand in the corresponding sRNA promoter (50 nts upstream of the transcription start site) or to the template strand within the transcribed region, yet discards all spacers that are prone to off-targeting (i.e., spacers with less than three mismatches to a second genomic locus and preceded by a 5′-TTV-3′ PAM). When multiple spacers are available, those with a preferred GC content (between 35 and 75%), annealing to the promoter or the 5′ portion of the targeted sRNA, or flanked by the preferred 5′-TTTV-3′ PAM (22) are prioritized. In addition, for each sRNA, our script attempts to design one array composed of three further, nonoverlapping guides, taking correct array folding (see above) into account. In cases when array design is unsuccessful—either because of an insufficient number of available spacers, or due to improper folding of all possible arrays—only gRNAs are outputted. As a result, the script delivers a list of sequences: for gRNAs, spacers flanked by homology regions for Gibson assembly, and for each array, six sequences comprising both strands of each repeat-spacer unit flanked by compatible 4-nt overhangs for CRATES (31) assembly.

Our script is freely available at https://github.com/gprezza/CRISPRi_tools and can be utilized to design libraries targeting any predefined gene set in *B*. *thetaiotaomicron* or any other species. Parameters such as PAM preference, strand-specificity, and array design are customizable, rendering the software suitable for versatile CRISPR applications. In the present context, a total of 577 gRNAs and 91 arrays were output by the script for the systematic knockdown of *Bacteroides* sRNAs. After cloning and conjugating the respective constructs into *B*. *thetaiotaomicron*, sequencing revealed that the vast majority (>99%) of the expected gRNAs and arrays were indeed contained in the resulting library. Most importantly, each of the 135 sRNAs was targeted in at least two library members, and only four sRNAs had fewer than the intended four gRNAs/arrays (*SI Appendix*, Fig. S4 *B* and *C*). This guide library therefore lends itself to systematic sRNA knockdown fitness screening.

### CRISPRi Screening Identifies BatR as a Modulator of *Bacteroides* Susceptibility to Bile Stress.

**Fig. 4.**
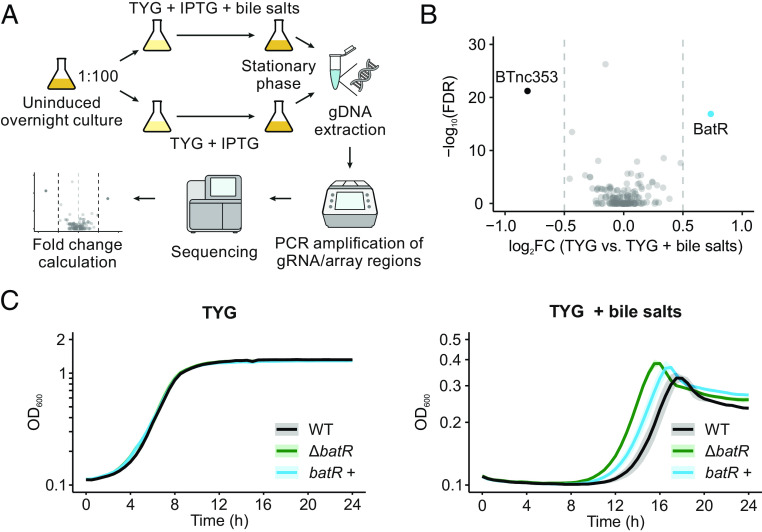
CRISPRi screening identifies an sRNA-associated bile stress phenotype. (*A*) Workflow of the CRISPRi screen. An overnight culture of the library is diluted 1:100 into fresh medium with IPTG, with or without 0.05 mg/mL of bile salts. After selection, the region of the genome containing the gRNAs/arrays is PCR-amplified from both subcultures and sequenced, thus enabling quantification of the library members and inferring a fitness score for each sRNA depletion under bile salt stress. (*B*) Volcano plot of log_2_FC vs. false discovery rate of the per-sRNA summarized data from the bile salt selection experiment. sRNAs with log_2_FC > 0.5 and FDR < 0.05 are highlighted. Two dashed vertical lines mark the −0.5 and +0.5 log_2_FC thresholds. A positive log_2_FC means that library members targeting the sRNA were on average enriched after growth in the presence of bile salts, a negative log_2_FC indicates average depletion in the same condition. (*C*) Growth of the wild-type, Δ*batR*, and *batR* complementation (*batR*+) strains in TYG medium without (*Left*) or with (*Right*) 0.05 mg/mL of bile salts. The colored lines refer to the means, and the shaded areas represent the SD of three biological replicates.

Bile acids, whose amphipathic nature poses continuous stress to bacterial membranes (32), are important factors shaping the composition of the intestinal microbiota. As a test case for the utility of our CRISPRi approach for identifying biologically relevant noncoding RNAs, we screened for sRNAs that affect *Bacteroides* fitness in the presence of bile salts. To this end, we subcultured three independent uninduced overnight cultures of the input library into fresh TYG medium containing 250 μM of IPTG, either with or without 0.05 mg/mL of bile salts. Once the cultures reached the stationary phase [~13.5 h if unstressed and 24 h in the presence of bile salts (10)], we harvested the bacteria, extracted their genomic DNA, and PCR-amplified the full CRISPRi locus. After high-throughput sequencing, we compared the relative abundance of the guides in the presence or absence of bile stress, thereby deriving the relative fitness of each library member. To infer the effects of individual sRNAs, we averaged the fitness scores over different guides targeting the same sRNA (Fig. 4*A*). This led to the prediction of two sRNAs whose silencing significantly affected *B*. *thetaiotaomicron* growth in the presence of bile salts (using an average fitness cutoff of |log_2_FC| > 0.5). Specifically, knockdown of BatR (formerly known as BTnc167 and here renamed to Bile acid tolerance Regulator for reasons to follow) or BTnc353 resulted in increased or decreased bacterial numbers under bile stress, respectively, as compared with the bulk of the library members (Fig. 4*B*).

To validate the phenotype derived from the CRISPRi screen, we generated a clean deletion mutant of BatR and cultured it in TYG medium with or without 0.5 mg/mL of bile salts. As compared with an isogenic wild-type strain, the Δ*batR* strain did not display any growth difference in normal TYG, yet showed earlier entry into the log phase and accumulated to higher OD when cultured in the presence of bile salts (Fig. 4*C*). This phenotype reflects the enhanced fitness observed in the CRISPRi screen. We constructed a BatR *trans*-complementation strain (*batR*+) by integrating the sRNA gene and its native promoter into the unrelated *tRNA^Ser^* locus in the Δ*batR* background. Despite the *batR*+ strain reaching only ~6% of the wild-type BatR level (*SI Appendix*, Fig. S5*A*), this low expression was sufficient to partially complement the bile phenotype (Fig. 4*C*). Taken together, these data support the specificity of the BatR-associated growth reduction during bile stress and provide proof of principle for our CRISPRi approach to identify sRNA-associated fitness phenotypes.

### BatR Is a Conserved sRNA Predicted to Regulate Cell Surface Genes.

**Fig. 5.**
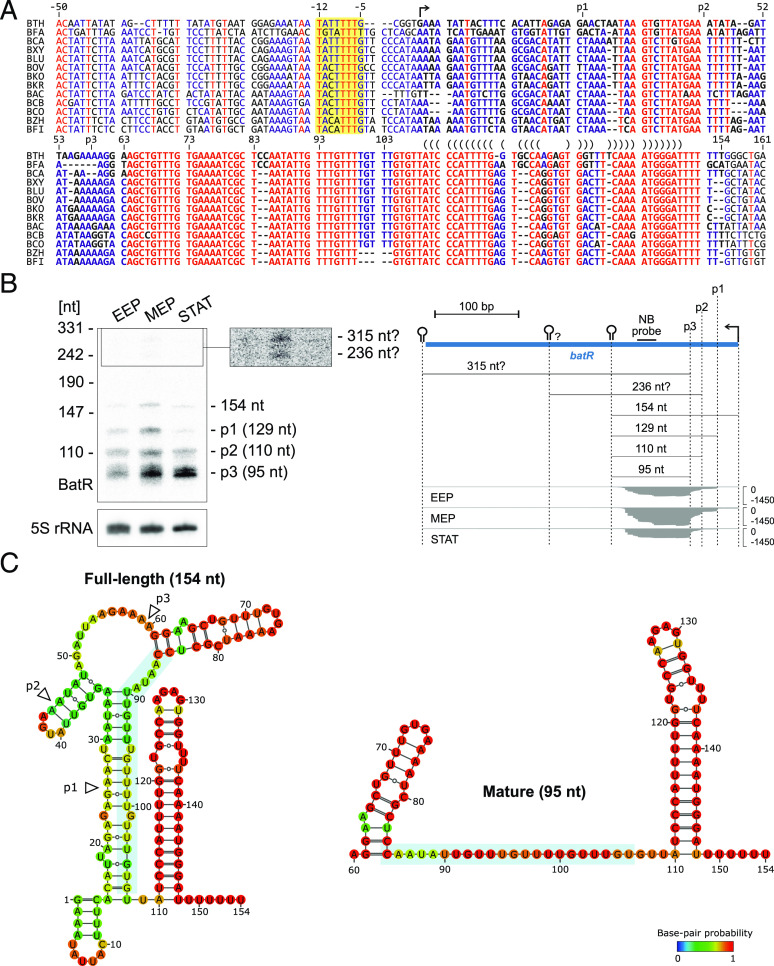
BatR is a conserved sRNA in *Bacteroides* spp. (*A*) Conservation of the *batR* genomic region spanning the four most prominent sRNA isoforms (see panel *B*). The −7 promoter box is highlighted in yellow, with the transcription start site indicated by an arrow. A bracket notation above the sequence marks the intrinsic terminator hairpin. BTH: *B*. *thetaiotaomicron*, BFA: *B*. *faecis*, BCA: *B*. *caecimuris*, BXY: *B*. *xylanisolvens*, BLU: *B*. *luhongzhouii*, BOV: *B*. *ovatus*, BKO: *B*. *koreensis*, BKR: *B*. *kribbi*, BAC: *B*. *acidifaciens*, BCB: *B*. *sp*. *CBA7301*, BCO: *B*. *congonensis*, BZH: *B*. *zhangwenhongii*, BFI: *B*. *finegoldii*. (*B*) *Left*: northern blot–based validation of BatR size and expression profile in rich medium. *Right*: RNA-seq-derived expression profile of the annotated *batR* locus in rich medium. All isoforms observed on the northern blot are labeled and their sizes are indicated. The previously annotated transcript contains two Rho-independent terminators that are marked with a hairpin symbol, and a possible third hairpin, additionally marked with a question mark. (*C*) Predicted secondary structure of full-length BatR (*Left*) or its shortest detected isoform (*Right*). Putative processing sites are indicated by white arrowheads and the seed region by a light blue background box. Secondary structures were predicted with RNAfold (33), using default parameters.

In light of the bile phenotype of the corresponding deletion mutant, we began to functionally characterize BatR. The sRNA itself and the corresponding –7 promoter box (20) are conserved in other *Bacteroides* species (Fig. 5*A*). Synteny analysis of the *batR* locus across *Bacteroides* spp. revealed its co-occurrence with uncharacterized transcriptional regulators and DNA-binding proteins (sigma factors, zinc finger proteins, and Lrp/AsnC ligand binding domain-containing proteins), while a coconservation with a set of isomerases, metallopeptidases, and methyltransferases was observed in most species other than *B*. *thetaiotaomicron* (*SI Appendix*, Fig. S5*B*). Assessment of our previously published transcriptomics data (10, 20) revealed that the sRNA is up-regulated in exponentially growing bacteria, during carbon starvation, and after exposure to the secondary bile acid deoxycholate (*SI Appendix*, Fig. S5*C*). While the predicted length of BatR inferred from RNA-seq data was 374 nts (20), northern blotting revealed a transcript close to this length to be generally of low abundance in the cell (Fig. 5 *B*, *Left*). Instead, a 154 nt-long transcript and three shorter isoforms thereof accumulated, with the latter ones potentially deriving from 5′ processing events (“p1,2,3” processing sites, Fig. 5 *B*, *Right*). Based on these observations, we reannotated BatR in *B*. *thetaiotaomicron* as the 154 nt isoform. The predicted secondary structure of BatR comprises a strong Rho-independent terminator at the 3′ end, a second strong hairpin in the central position, and a structured region near the 5′ end (Fig. 5 *C*, *Left*). Processing at any of the three sites would likely render the region between position 86 and 109 single-stranded (Fig. 5 *C*, *Right*) and hence, available for pairing with complementary stretches in target mRNAs.

**Fig. 6.**
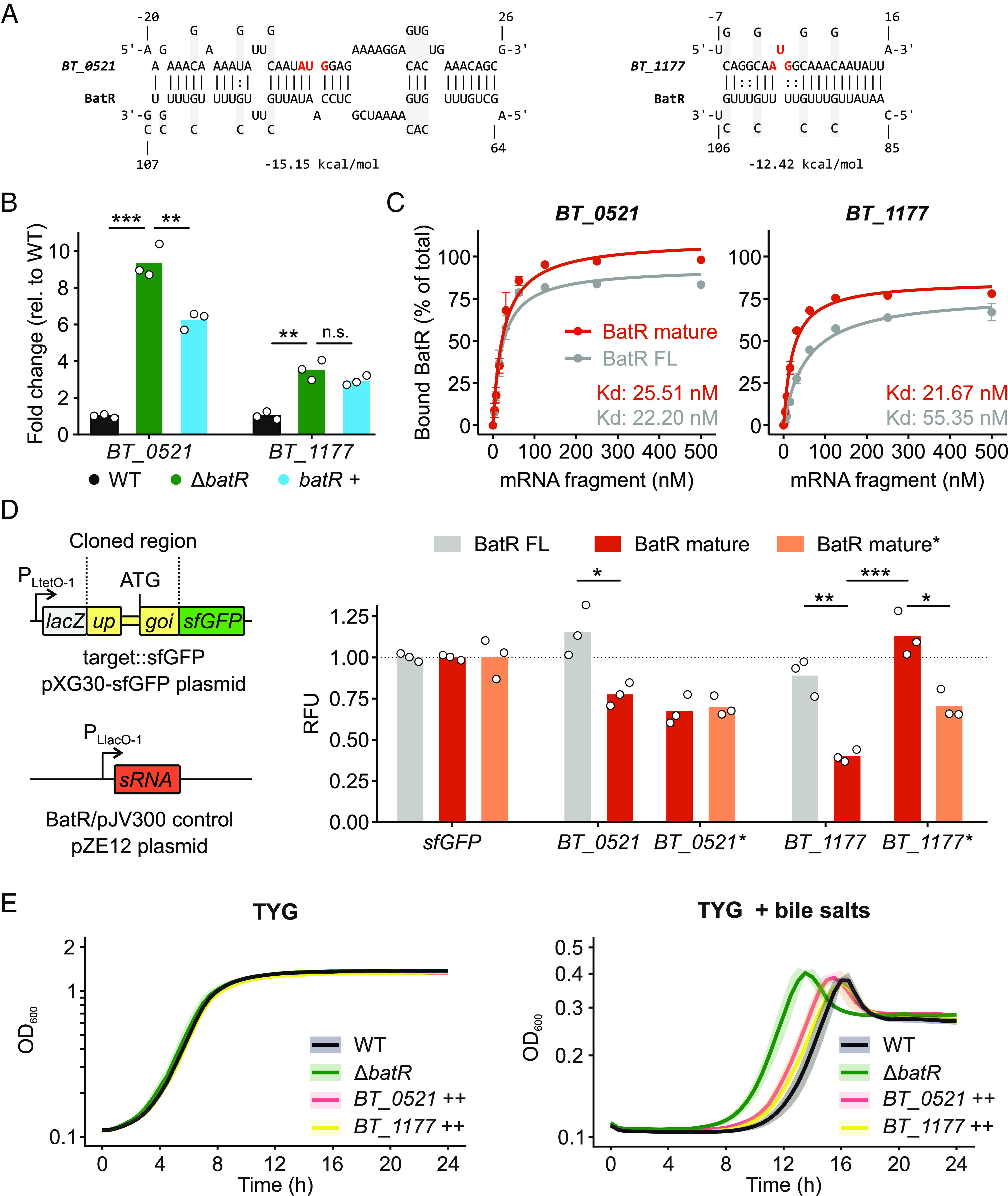
BatR represses cell surface–related genes. (*A*) Interaction site between BatR and two putative mRNA targets as predicted by CopraRNA. The start codon is highlighted in red font. Bases that were mutated in the corresponding GFP reporter experiments (see panel *D*) are highlighted with gray background, with the mutated nucleotides above or below the wild-type sequence. (*B*) qRT-PCR measurement of the levels of two BatR target candidates in the wild-type, Δ*batR*, and *batR* complementation (*batR*+) strains. Dots denote the values of the individual biological replicates. ***P-value < 0.001, **P-value < 0.01, n.s.: *P*-value > 0.05; unpaired *t* test. (*C*) Quantification of the fraction of bound, radiolabeled BatR at increasing concentrations of *BT_0521* (*Left*) or *BT_1177* (*Right*) from EMSAs shown in *SI Appendix*, Fig. S6*C*. Error bars represent the SD of two biological replicates. FL: full-length. (*D*) Translational reporter experiments with the predicted BatR interaction regions fused to superfolder GFP (sfGFP). *Left*: schematic of the two plasmids used for the experiment. In pXG30-sfGFP-based plasmids (*Top*), the N terminus of the gene of interest (“goi”) and the C terminus of its upstream gene in the same operon (“up”) are cloned in-frame between the *lacZ* and *sfGFP* cassettes. The constitutive promoter P_LtetO-1_ drives transcription of the resulting dicistronic operon. pZE12-based plasmids (*Bottom*) constitutively express BatR (full-length or mature isoform) or a nonsense control RNA (plasmid pJV300). *Right*: GFP signal intensity of strains harboring different plasmid combinations, normalized to the signal of the strain carrying the respective mRNA fusion and pJV300 plasmids, and second, to the strain carrying the pXG30-sfGFP and respective pZE-BatR plasmids. An asterisk indicates a mutated BatR or target variant (see panel *A*). The bars represent the average of three biological replicates, which are individually plotted as circles. ***P-value < 0.001, **P-value < 0.01, *P-value < 0.05; unpaired *t* test. (*E*) Growth of wild-type, Δ*batR*, *BT_0521* overexpression (*BT_0521*++), and *BT_1177* overexpression (*BT_1177*++) *B*. *thetaiotaomicron* strains in TYG medium without (*Left*) or with (*Right*) 0.05 mg/mL of bile salts. The colored lines refer to the means and the shaded areas represent the SD of three biological replicates.

An in-silico target search using the CopraRNA tool (34) predicted that BatR binds the translation initiation region of a set of mRNAs encoding proteins with a predicted role in cell-surface modulation (Fig. 6*A*, *SI Appendix*, Fig. S6*A*, and Dataset S5). Among the top ten target candidates (based on minimal free energy calculations upon BatR annealing), we picked four for further validation, namely *BT_0521* (partially homologous to the polysaccharide capsule synthesis protein CpsI from *Streptococcus iniae*), *BT_1177* (encoding an LPS biosynthesis protein), *BT_1195* (encoding a peptidoglycan transglycosylase), and *BT_3849* (encoding the LPS-assembly protein LptD). We collected total RNA from wild-type, Δ*batR*, and *batR*+ strains cultured in TYG medium to mid-exponential phase (OD_600_ = 2). The ensuing qRT-PCR analysis revealed derepression of *BT_0521* (~eightfold) and *BT_1177* (~fivefold) in the absence of BatR, while complementation with full-length BatR partially restored *BT_0521*—but not *BT_1177*—repression (Fig. 6*B*). Steady-state mRNA levels of the other two target candidates remained unchanged by BatR (*SI Appendix*, Fig. S6*B*).

Electrophoretic mobility shift assays (EMSAs) using radiolabeled full-length (154 nt) BatR or its 95 nt isoform demonstrated direct binding to the 5′ regions of each of the above four predicted target candidates (*SI Appendix*, Fig. S6*C*). In line with our hypothesis that the sRNA’s seed region is partially occluded in full-length BatR but opens up upon processing, the dissociation constant (K*_d_*) and/or the fraction of bound sRNA at saturation were superior for the mature isoform as compared with full-length BatR (Fig. 6*C* and *SI Appendix*, Fig. S6*D*). The lower affinity of the full-length sRNA to *BT_1177* as compared to *BT_0521* (Fig. 6*C*) provides a possible explanation for the more efficient *trans*-complementation in case of the latter target under conditions when BatR steady-state levels are likely rate-limiting (due to the low expression in the complementation strain; *SI Appendix*, Fig. S5*A*).

### Validation of BatR Targets In Vivo.

To provide confirmation that regulation of the four BatR target candidates occurs in vivo, we made use of an established dual-plasmid fluorescent reporter system in *E*. *coli* (35, 36). Since all four candidates are intraoperonic, the respective target regions were cloned into the pXG30-sfGFP vector, which contains a dicistronic operon structure composed of the *lacZ* and *superfolder GFP* (*sfGFP*) genes (Fig. 6 *D*, *Top Left*) (36). BatR (either the 154-nt full-length or the 95-nt mature isoform; *SI Appendix*, Fig. S7*A*) was coexpressed from a second plasmid [pZE12 (37)] (Fig. 6 *D*, *Bottom Left*). Note that in the heterologous *E*. *coli* system, no processing of full-length BatR into the 95-nt mature isoform occurred (*SI Appendix*, Fig. S7*A*). With this experimental setup, we observed repression of all four translational GFP fusions upon expression of the mature BatR (Fig. 6 *D*, *Right* and *SI Appendix*, Fig. S7*B*). Importantly, full-length BatR led to none or limited regulation, corroborating our hypothesis that the secondary structure of the longer sRNA isoform hampers target binding by masking the seed sequence (Fig. 5 *C*, *Left*). In addition, mutation of critical bases (indicated by gray shading in Fig. 6*A* and *SI Appendix*, Fig. S6*A*) in the predicted interaction region of the *BT_1177* and *BT_1195* reporters compromised regulation (Fig. 6 *D*, *Right* and *SI Appendix*, Fig. S7*B*). BatR carrying compensatory mutations (gray shading in Fig. 6*A* and *SI Appendix*, Fig. S6*A*) appeared to be toxic in *E*. *coli*, as we only obtained clones carrying additional, unwanted mutations in either the promoter or seed region of the pZE12-BatR* construct. Of those, we selected a clone carrying a single G deletion at position –12 in the promoter that, as a consequence, expressed the mutated mature BatR at ~5% of the wild-type level (*SI Appendix*, Fig. S7*A*) and was tolerated by *E*. *coli* cells. Despite this reduced level, mutant BatR partially restored regulation of the mutated *BT_1177* and *BT_1195* reporters and repressed the mutated *BT_0521* and *BT_3849* reporters equally well as did its—more abundant—wild-type counterpart (Fig. 6 *D*, Right and *SI Appendix*, Fig. S7*B*). Taken together, our combined data suggest BatR as a posttranscriptional regulator of *Bacteroides* cell surface structure, with implications for the bacteria’s tolerance of bile stress, all based on genome-wide screening of sRNAs using an efficient CRISPRi system we established in *B*. *thetaiotaomicron*.

## Discussion

Well-studied bacteria, such as the species belonging to Enterobacteriaceae, employ sRNAs to cope with stresses and changing environmental conditions (8). Yet to what extent obligate anaerobic bacteria of the intestinal microbiota depend on sRNAs to thrive in the fluctuating niches of the gut remains an understudied branch of microbiome research. The Bacteroidetes represent a predominant phylum of human gut microbes (4). Recently, studies from us and others identified sRNAs in different gut Bacteroidetes species and shed light on functional aspects of a few hand-selected candidate sRNAs (reviewed in ref. 9). However, the phenotypic consequences of these sRNAs’ activities have largely remained obscure. Only for a single *Bacteroides* sRNA—termed MasB—could a loss-of-function phenotype be identified (10). Guided by TIS data, we found *masB*-deficient *B*. *thetaiotaomicron* mutants to have an enhanced tolerance of tetracyclines. In general terms, however, TIS—like any other random perturbation approach—biases against mutants of short genes, e.g., those of sRNAs. Thus, to systematically dissect the functional implications of these cellular regulators, tailored assays are required.

Acknowledging this need, we here introduced CRISPRi to sRNA research in *Bacteroides*. In light of the AT-rich nature of *Bacteroides* spp. genomes, we based our screen on the 5′-TTV-3′ PAM-recognizing nuclease Pb2Cas12a. The ensuing targeting space extension within the intergenic sRNA repertoire of *B*. *thetaiotaomicron* compared to that of SpCas9 allowed for more stringent spacer design rules, including “soft” gRNA parameters like optimal GC content and minimal risk of off-targeting. Our library design script is customizable, allowing users among others to select a desired PAM, and will hence be useful to construct libraries for other custom gene sets in virtually any bacterial species.

As a compelling test case for the current study, we harnessed CRISPRi to assess the fitness of *B*. *thetaiotaomicron* sRNA knockdown strains during bile stress. As a result, our screen proposed the sRNA BatR to reduce *B*. *thetaiotaomicron* bile stress resilience. We confirmed this sRNA to delay *B*. *thetaiotaomicron* outgrowth in the presence of bile salts. According to our target identification, BatR interacts with the 5′ regions of four mRNAs that encode for proteins involved in the modulation of the cell surface. Two of the BatR target mRNAs that we validated in this study, namely *BT_1177* and *BT_3849*, play roles in LPS biogenesis. The remaining targets (*BT_0521* and *BT_1195*) code for predicted homologs of an acyl transferase involved in capsule synthesis (38) and of *mgtA*, a transglycosylase linked to peptidoglycan biosynthesis (39). Alterations at their surface can change the ability of bacteria to withstand bile stress (40). We thus hypothesized that the dysregulated levels of surface-related BatR target proteins would contribute to the observed bile phenotype of the sRNA deletion mutant, and indeed, overexpression of *BT_1177* partially phenocopied the Δ*batR* growth advantage during bile stress (Fig. 6*E*).

For two targets—*BT_0521* and *BT_1177*—BatR binding resulted in reduced transcript levels in vivo, while the steady-state mRNA levels of the two remaining candidates were not affected by the sRNA in this experimental condition (midexponential growth in rich medium). Conversely, we did observe BatR-mediated repression of the translational of each target candidate, arguing that all four cell surface genes are bona fide BatR targets. Therefore, and in light of the BatR binding sites overlapping with the translation initiation regions of most of its target mRNAs, we propose that the primary mechanism of BatR is to block ribosomal assembly on these targets, which may in some cases (*BT_0521*, *BT_1177*) be accompanied by the destabilization of the translationally repressed mRNAs (41). In any case, BatR seems to rely on 5′ end maturation for efficient target interaction. Such a processing-dependent liberation of an sRNA’s seed region is reminiscent of proteobacterial sRNAs, such as *Salmonella* ArcZ (42) and *Campylobacter* CJnc190 (43). While maturation of these sRNAs is catalyzed by RNase E or RNase III, respectively, the *Bacteroides* nuclease(s) responsible for BatR processing remain(s) elusive. However, given that BatR processing did not occur in the heterologous host *E*. *coli* (*SI Appendix*, Fig. S7*A*), the *Bacteroides* RNase in question is likely not conserved in Enterobacteriaceae. In light of its associated loss-of-function phenotype, the relevance of its target genes for *Bacteroides* cell surface structure, and the implied, unusual sRNA biogenesis/maturation, BatR arises as a prime candidate for follow-up mechanistic and functional studies.

Our screen further proposed the sRNA BTnc353 to increase *Bacteroides* resilience to bile stress. While this prediction was not followed up experimentally in the current study, we note that two computationally predicted mRNA targets of BTnc353—namely *BT_4053* and *BT_4721* (Dataset S6)—were down- or up-regulated, respectively, in previous data when *B*. *thetaiotaomicron* was bile-stressed (10). BT_4053 is part of the family of mechanosensitive channels, i.e., proteins that sense membrane tension (44), which arises among others during bile stress. BT_4721 is likely an anti-sigma factor, acting in concert with the cognate extracytoplasmic function (ECF) sigma factor BT_4720, encoded upstream in the same operon (45). In this context, it appears interesting to test for a potential role of BT_4720/21 as an ECF sigma/anti-sigma factor pair governing the transcription of bile stress-response genes in *B*. *thetaiotaomicron*, and of BTnc353 as a putative posttranscriptional regulator of such a transcriptional control system.

In the future, our CRISPRi approach may be used to screen for sRNA functions under a variety of further growth conditions. Of specific interest to *B*. *thetaiotaomicron* biology are experimental conditions mimicking the natural environment of these gut microbes, such as growth in the presence of carbon sources derived from the host or its diet (46). Beyond simplistic in vitro settings, the CRISPRi library could be deployed for colonization experiments of cell culture or mouse models. Such in vivo applications typically involve growth over extended time spans and will therefore require careful planning of the experimental setup. For example, it will be essential to ensure continuously high concentrations of the nuclease inducer in order to maintain selective pressure throughout the experiment [note that constitutive expression of Cas nucleases is generally not an option due to the resulting toxic effects observed in multiple bacterial species (47–51)]. This might be achieved through regularly replenishing fresh IPTG in the medium (for cell culture–based experiments) or drinking water (for animal models). Of note, in case of the latter, colonization with a small, predefined CRISPRi library likely overcomes the bottleneck effects often hampering in vivo screens based on genome-wide transposon insertion libraries composed of hundreds of thousands of individual mutants (14).

Beyond targeting noncoding RNAs, the resources described in this work add to the functional toolset available for *Bacteroides* spp. and shall be useful for various other applications. Small proteins, for example, are prevalent across the bacterial phylogenetic tree and have also been annotated in *Bacteroides* spp. (15, 52). Suffering from similar limitations associated with sRNAs, small protein mutants tend to be underrepresented in classical transposon libraries. In contrast, a targeted CRISPRi approach analogous to the one described here would allow researchers to systematically screen for small protein-associated phenotypes. Besides, our work lays the ground for future genetic interaction screens (53) in which CRISPRi constructs targeting defined gene sets would be introduced in a preexisting mutant background. Alternatively, our approach could be adapted for CRISPR activation (16), wherein a Cas nuclease is fused to a transcriptional activator, resulting in the specific upregulation of targeted genes. The functional insights gained from these and related studies would help improve our fundamental understanding of the bacterial members of the gut microbiota.

## Materials and Methods

Lists of strains, plasmids, oligonucleotides, and media used in this work are available in Dataset S1. Python scripts for PAM frequency determination and CRISPRi library design are deposited at https://github.com/gprezza/CRISPRi_tools. A comprehensive description of materials and methods is provided in *SI Appendix*, *Materials and Methods*. Briefly, cloning was performed in Gibson assembly-like reactions using the NEBuilder® HiFi DNA Assembly kit (NEB, E2621), except for the assembly of arrays, which was accomplished through CRATES (31). Genomic DNA was obtained from CRISPRi strains using a standard phenol-chloroform-based extraction method and sequenced in paired-end mode, 150 bp reads. Differential abundance analysis of library members was calculated with edgeR (3.32.1). RNA extraction, northern blotting, qRT-PCR, and GFP-fusion reporter assays were performed as described previously (20, 35, 36). For the latter, the fluorescence intensity of 50,000 events was measured on an Agilent NovoCyte Quanteon flow cytometer.

## Supplementary Material

Appendix 01 (PDF)

Dataset S01 (XLSX)

Dataset S02 (XLSX)

Dataset S03 (XLSX)

Dataset S04 (XLSX)

Dataset S05 (XLSX)

Dataset S06 (XLSX)

## Data Availability

Sequencing data have been deposited in the National Center for Biotechnology Information Gene Expression Omnibus database (GSE235620) (54). Plasmids AWP-029 and AWP-031 have been deposited at Addgene (https://www.addgene.org) under the IDs 213966 and 213967, respectively.
