# Supplementary material for: CRISPR-based screening of small RNA modulators of bile susceptibility in Bacteroides thetaiotaomicron
Source: Proc Natl Acad Sci U S A. 2024 Jan 31;121(6):e2311323121. doi: 10.1073/pnas.2311323121 (PMC10861873; doi:10.1073/pnas.2311323121)
Supplement: Supplementary file 1 — Appendix 01 (PDF) [file pnas.2311323121.sapp.pdf]

PNAS

# Supporting Information for

# CRISPR-based screening of small RNA modulators of bile susceptibility in *Bacteroides thetaiotaomicron*

Gianluca Prezza[1], Chunyu Liao[1], Sarah Reichardt[1], Chase L. Beisel[1,2], Alexander J. Westermann[1,3,4,*]

[1]Helmholtz Institute for RNA-based Infection Research (HIRI), Helmholtz Centre for Infection Research (HZI), Würzburg, D-97080, Germany

[2]Medical Faculty, University of Würzburg, Würzburg, D-97080, Germany

[3]Institute of Molecular Infection Biology (IMIB), University of Würzburg, Würzburg, D-97080, Germany

[4]Department of Microbiology, Biocentre, University of Würzburg, Würzburg, D-97074, Germany

[*]Correspondence: alexander.westermann@uni-wuerzburg.de

**This PDF file includes:**

Table of contents
Figures S1 to S7
Materials and methods
Legends for Datasets S1 to S6
SI References

**Other supporting materials for this manuscript include the following:**

Datasets S1 to S6

## 1 TABLE OF CONTENTS

1 TABLE OF CONTENTS .......... 2
2 SUPPLEMENTARY FIGURES .......... 3
3 MATERIAL AND METHODS .......... 13
Bacterial strains and culture conditions .......... 13
PAM frequency determination .......... 13
Nuclease screening by in vitro transcription and translation (TXTL) .......... 13
Western blotting .......... 13
Cloning of nucleases, gRNAs, and arrays .......... 13
Luciferase assay .......... 14
RNA extraction, northern blotting, qRT-PCR .......... 15
Design and cloning of the CRISPRi library .......... 15
CRISPRi screen under bile salt stress .......... 16
Sequencing and data analysis for the CRISPRi fitness screen .......... 17
Construction of batR deletion and complementation mutants and of BT_0521 or BT_1177 overexpression strains .......... 17
B. thetaiotaomicron growth curves .......... 18
sRNA homolog search, target prediction, and synteny analysis .......... 18
In-vitro transcription and 5′ end labelling .......... 18
Electrophoretic mobility shift assay (EMSA) .......... 18
GFP reporter assay in E. coli TOP10 .......... 19
4 LEGENDS FOR DATASETS S1 to S6 .......... 20
5 SI REFERENCES .......... 21

## 2 SUPPLEMENTARY FIGURES

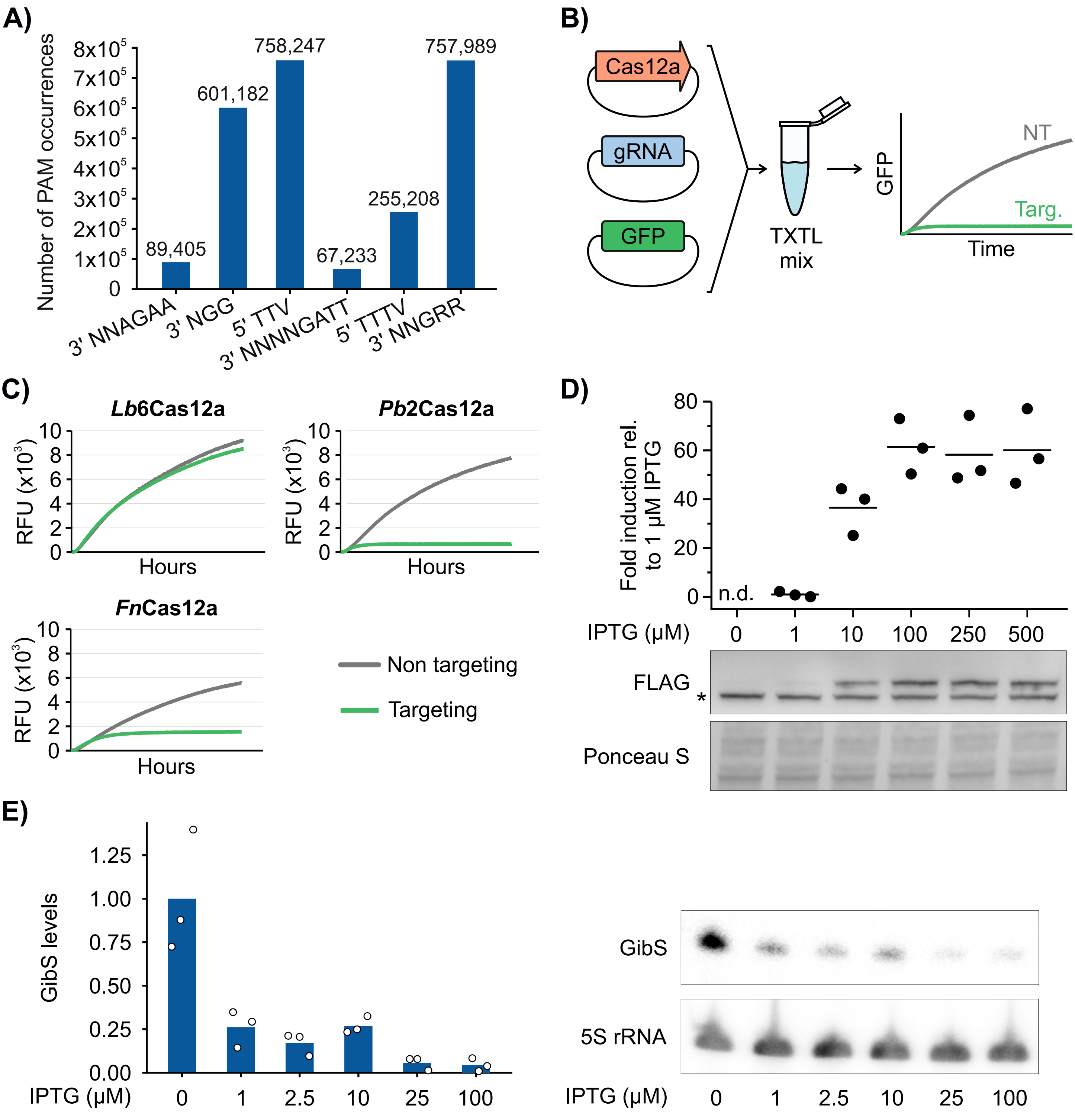

**Supplementary Figure S1**

A) Frequency of different PAMs within the entire genome of *B. thetaiotaomicron* VPI-5482. Related to Fig. 1A.

B) Scheme of the TXTL assay. A plasmid encoding GFP was co-expressed in a TXTL reaction with a Cas12a ortholog and a CRISPR array comprising either a gRNA targeting GFP or a non-targeting control. GFP  luorescence emission was recorded over time. A lower emission of the targeting array (Targ, green) versus the non-targeting one (NT, grey) indicate effective cleavage of the GFP coding sequence by Cas12a.

C) Plasmid clearance assay using three Cas12a orthologs. Relative  luorescence units (RFU) are plotted over time for each separate reaction (n=1).

D) Identi ication of the optimal IPTG concentration for induced expression of dPb2Cas12a. An overnight culture of a strain encoding dPb2Cas12a expressed under an IPTG-inducible promoter was subcultured 1:100 in fresh medium containing the indicated IPTG concentration. Top: quanti ication of dPb2Cas12a levels in three biological replicates, with a line representing the mean value. Bottom: representative western blot image and Ponceau S staining as loading control. An asterisk next to the blot indicates an unspeci ic band.

E) Correlation between dPb2Cas12a induction level and target knockdown ef iciency. Left: measurement of the levels of the GibS sRNA upon its knockdown (as introduced in Fig. 3) with dPb2Cas12a induced with increasing IPTG concentrations. Right: representative northern blot of three biological replicates.

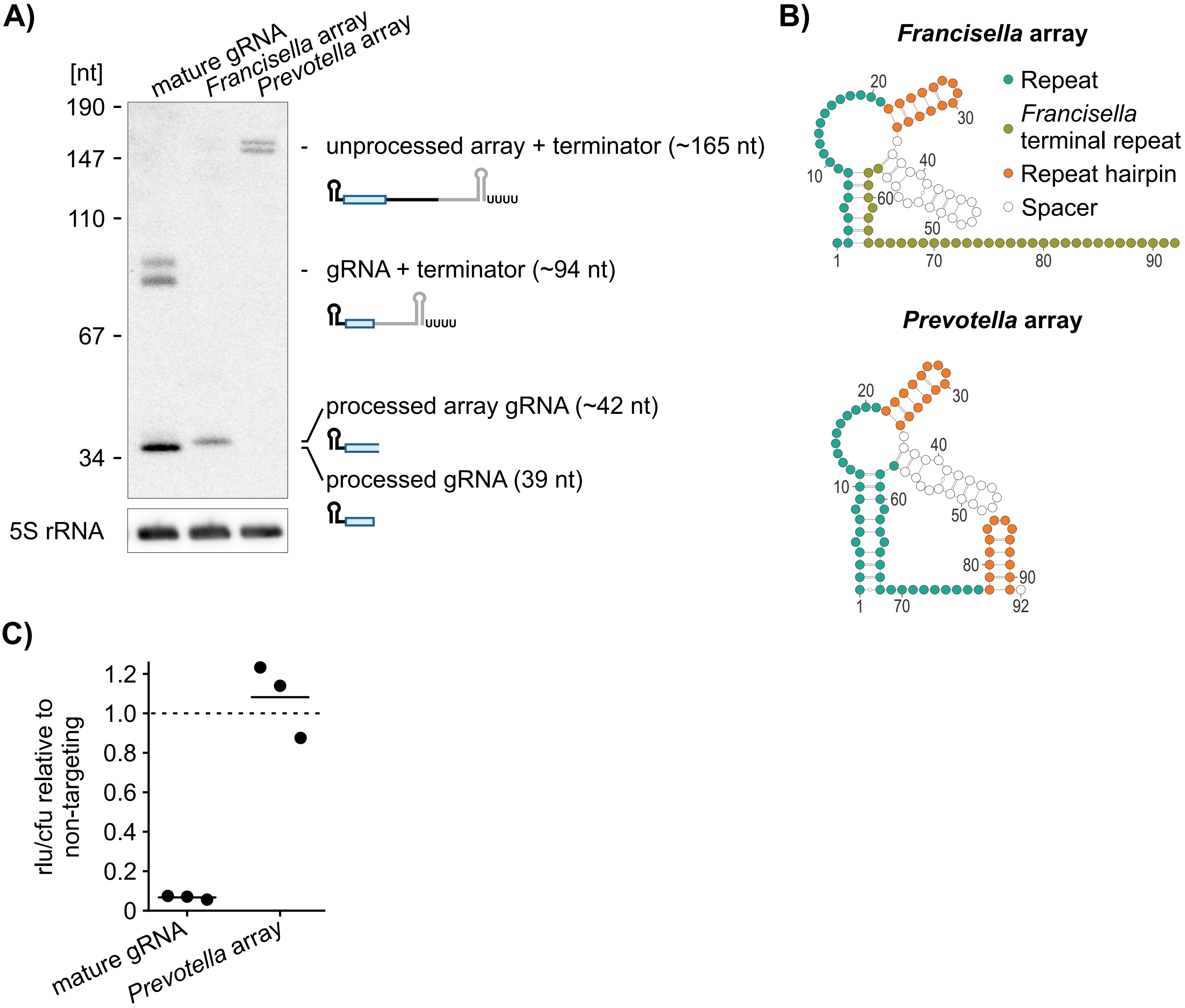


**Supplementary Figure S2**

A) Northern blot detection of processed and precursor gRNA transcripts in the strains shown in Fig. 2C and Supplementary Fig. S2C in mid-exponential phase (OD600 = 2.0; ~6.5 h). The probe used is complementary to the spacer sequence. The E. coli *rnpB* terminator hairpin following the array/gRNA sequences gets post-transcriptionally removed . The blot image is representative of three biological replicates.

B) Prediction of the secondary structure of the arrays used in Supplementary Fig. S2A with RNAfold . Correct formation of the repeat hairpin required for gRNA maturation  is highlighted in orange.

C) Knockdown of luciferase expression by dPb2Cas12a with the same spacer either expressed as a gRNA (mature gRNA) or surrounded by *Prevotella* repeats within a CRISPR array-like transcript. The data of the mature gRNA are the same as those shown in Fig. 2A for “p_T2”. Values are relative to the levels observed in a strain with a non-targeting spacer, encoded either as gRNA or as an array with *Prevotella* repeats (n=3).

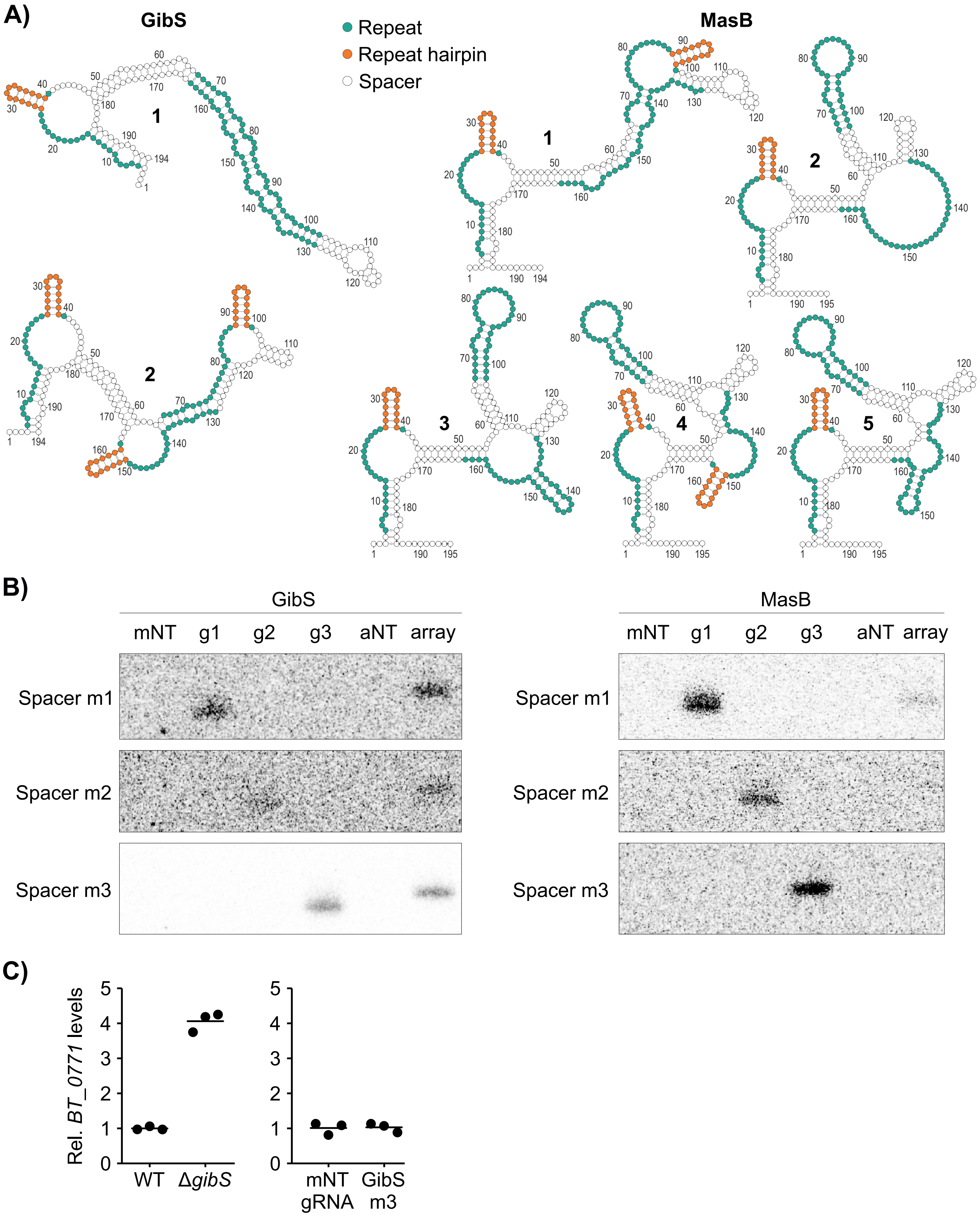
A)
GibS
MasB
Repeat
Repeat hairpin
Spacer
B)
GibS
mNT g1 g2 g3 aNT array
MasB
mNT g1 g2 g3 aNT array
Spacer m1
Spacer m2
Spacer m3
C)
Rel. BT_0771 levels
WT
ΔgibS
mNT gRNA
GibS m3

**Supplementary Figure S3**

A) Predictions of the secondary structure of the GibS- and MasB-targeting arrays. The ive structures with the lowest minimal free energy as predicted by mfold are shown, with a number next to each structure marking the rank position from lowest to highest predicted minimal free energy. Only two structures were predicted for the GibS array. Repeat sequences are highlighted in green, correctly formed terminal hairpins in orange.

B) Detection by northern blotting of processed gRNA levels in strains expressing stand-alone gRNAs or the same spacers encoded in an array. For the corresponding 5S rRNA signal (loading control), see Fig. 3A. The probes used in the experiment anneal to each spacer sequence. Images are representative of two biological replicates.

C) qRT-PCR measurements of *BT_0771* levels in Δ*tdk* (WT), Δ*tdk*Δ*gibS* (ΔgibS) or in bacteria harboring the CRISPRi vector with a non-targeting (gNT gRNA) or GibS-targeting (GibS g3) gRNA. Values shown are relative to Δtdk or the non-targeting gRNA, respectively. A line represents the mean value.

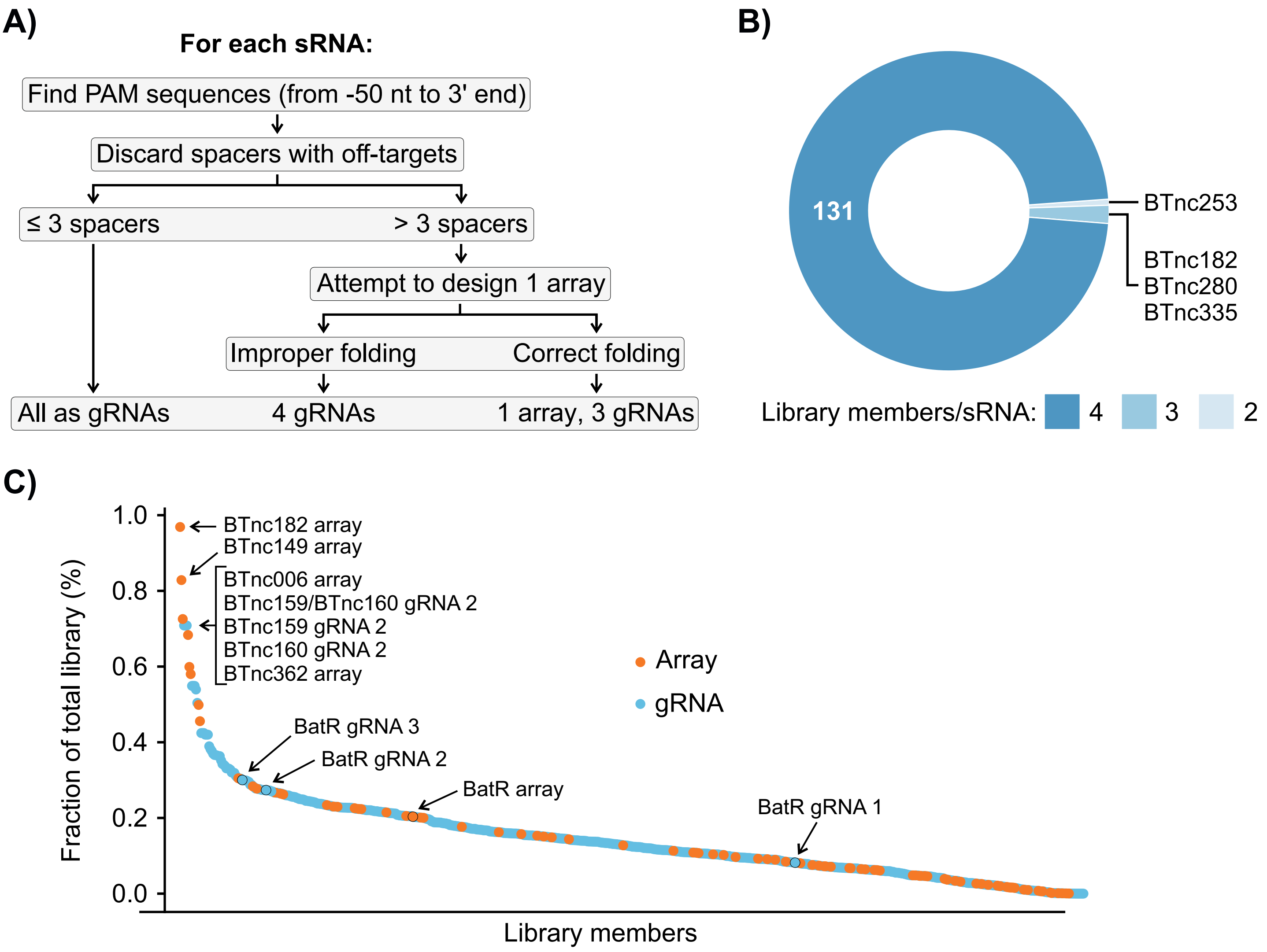


**Supplementary Figure S4**

A) Flow chart of the script used to design the gRNAs/arrays targeting intergenic sRNAs of *B. thetaiotaomicron*. PAM sequences are identi ied for each sRNA in between 50 nt upstream of the transcription start site and the 3′ end of the transcript. Off-targeting spacers are discarded and gRNAs/arrays are designed. Arrays that are not predicted to form a hairpin at the 3′ end of each repeat are discarded. As an output, the script proposes maximally four targeting constructs per sRNA.

B) Representation of the sRNA knockdown strains present in the  inal CRISPRi library.

C) Distribution of each gRNA or array in the library as fraction of the library size. The most abundant library members, as well as those targeting BatR are labeled.

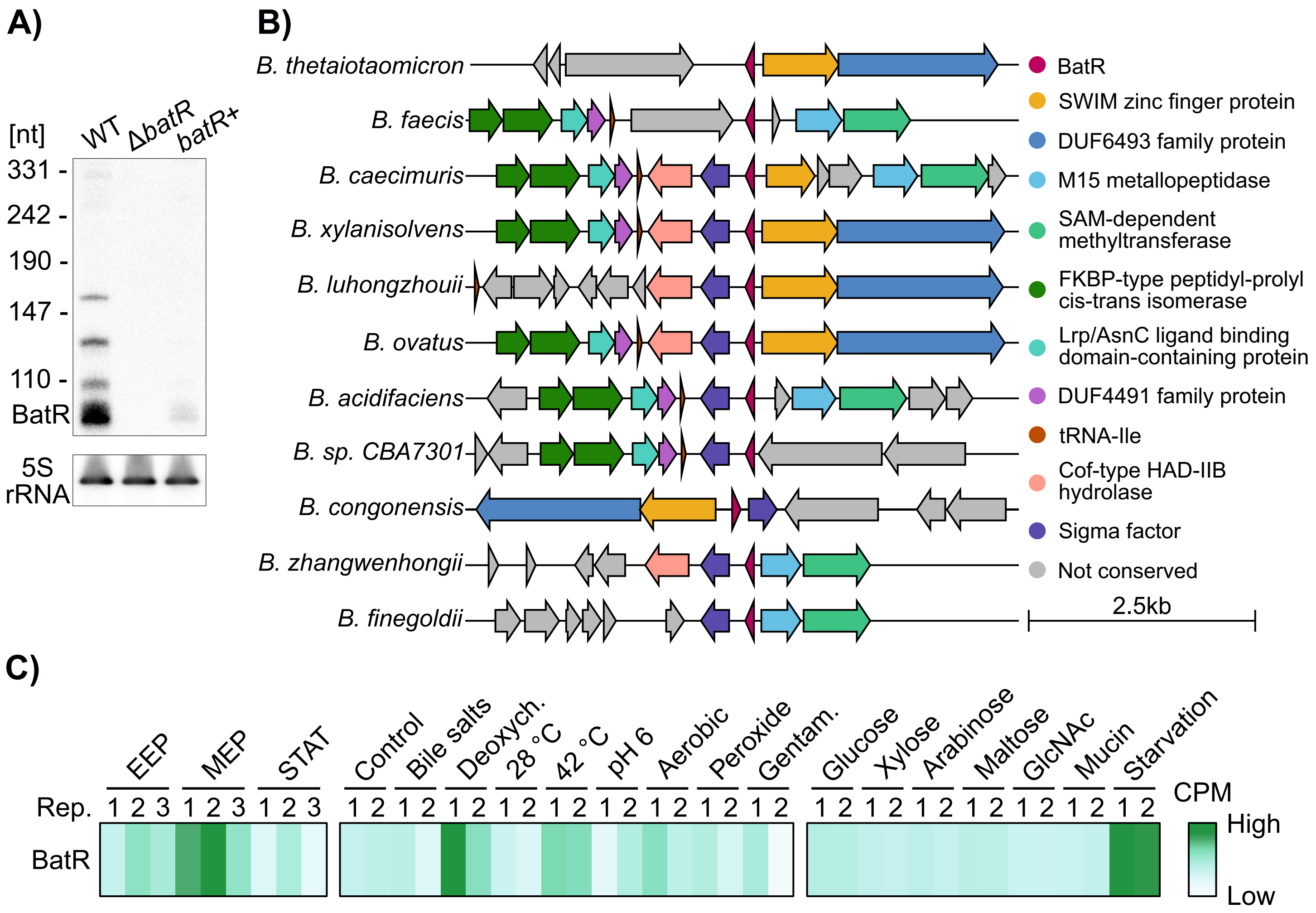


**Supplementary Figure S5**

A) Detection of BatR via northern blot in the wild-type, Δ*batR*, and *batR*+ *B. thetaiotaomicron* strains grown to mid-exponential phase (OD600 = 2.0). The 5S rRNA was probed for as a loading control. The blot shown is representative of three biological replicates.

B) Synteny analysis of BatR homologs across *Bacteroides* spp. For each strain, the 5,000 nts upstream and downstream of the batR gene are plotted. Genes within these regions are represented as arrows and colored according to their function.

C) Abundance (z-score of CPM values) of BatR during growth in rich medium (EEP: early exponential phase, MEP: mid-exponential phase, STAT: stationary phase), after exposure to different stressors or while feeding on de ined carbon sources.

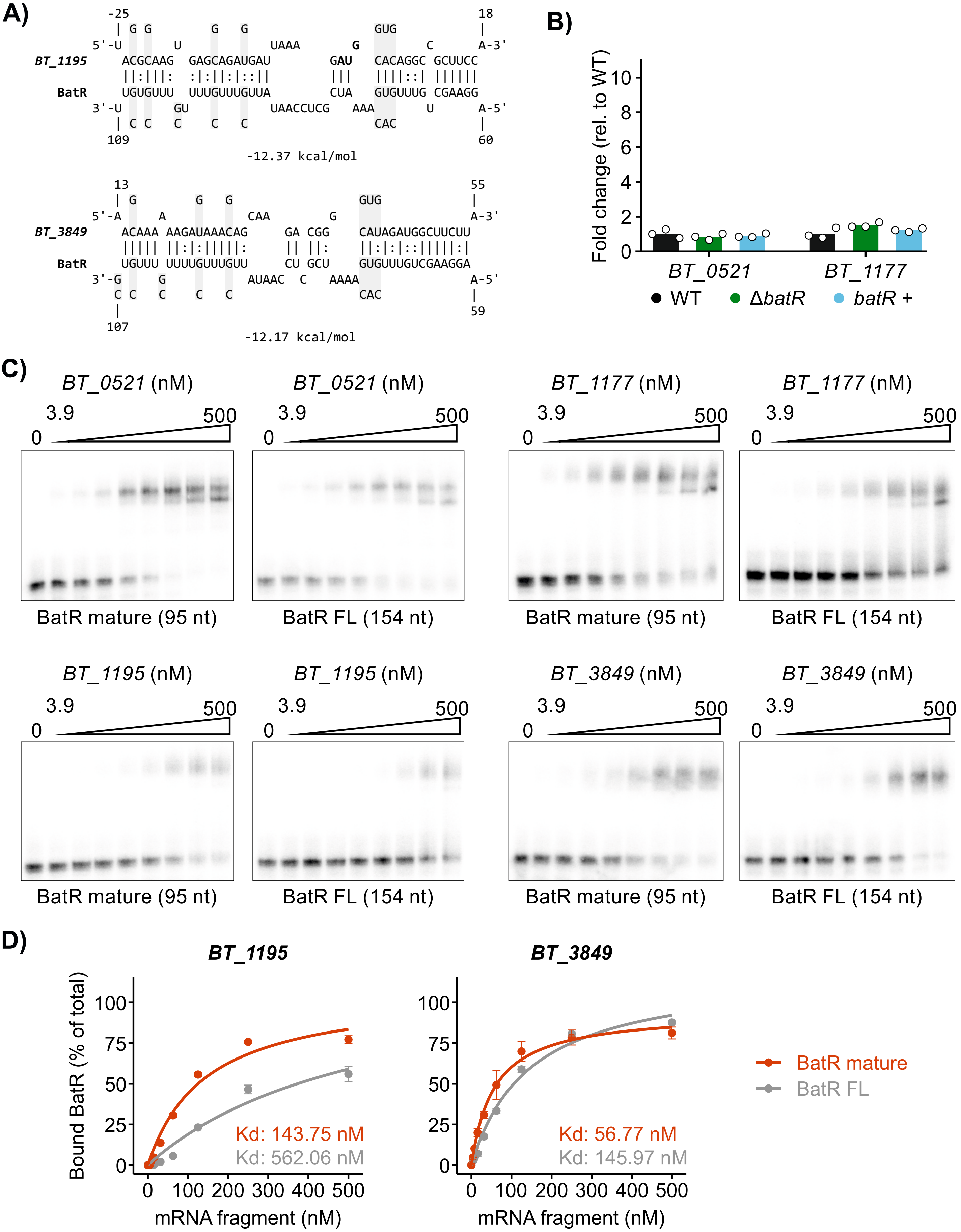
A)
-25
18
5'-U
A-3'
BT_1195
ACGCAAG GAGCAGAUGAU
GAU CACAGGC GCUUCC
BatR
UGUGUUU UUUGUUUGUUA
CUA GUGUUUG CGAAGG
3'-U
A-5'
109
60
-12.37 kcal/mol
13
55
5'-A
A-3'
BT_3849
ACAAA AAGAUAAACAG
GA CGG
CAUAGAUGGCUUCUU
BatR
UGUUU UUUUGUUUGUU
CU GCU
GUGUUUGUCGAAGGA
3'-G
A-5'
107
59
-12.17 kcal/mol
B)
Fold change (rel. to WT)
BT_0521
BT_1177
WT
ΔbatR
batR +
C)
BT_0521 (nM)
BT_1177 (nM)
BT_1195 (nM)
BT_3849 (nM)
3.9
500
0
BatR mature (95 nt)
BatR FL (154 nt)
D)
BT_1195
BT_3849
Bound BatR (% of total)
mRNA fragment (nM)
Kd: 143.75 nM
Kd: 562.06 nM
Kd: 56.77 nM
Kd: 145.97 nM
BatR mature
BatR FL

**Supplementary Figure S6**

A) Predicted interaction between BatR and the two putative mRNA targets *BT_1195* and *BT_3849*. Bases in the target gene that were mutated in the corresponding GFP reporters (see Supplementary Fig. S7B) are highlighted with gray background, with the mutated nucleotides above or below the wild-type sequence.

B) qRT-PCR measurement of the levels of *BT_1195* and *BT_3849* in the wild-type, Δ*batR*, and *batR* complementation (*batR*+) strains.

C) EMSA of BatR and 5′ fragments of four predicted mRNA targets encompassing the predicted interaction sites (see Fig. 6A and Supplementary Fig. S6A). BatR at a constant (4 nM) concentration was mixed with increasing (0 to 500 nM) concentrations of each mRNA fragment. Each gel is representative of two replicates. FL: full-length.

D) Quanti ication of the fraction of bound BatR at increasing concentrations of *BT_1195* (left) or *BT_3849* (right). Error bars represent standard deviation of two biological replicates. FL: full-length.

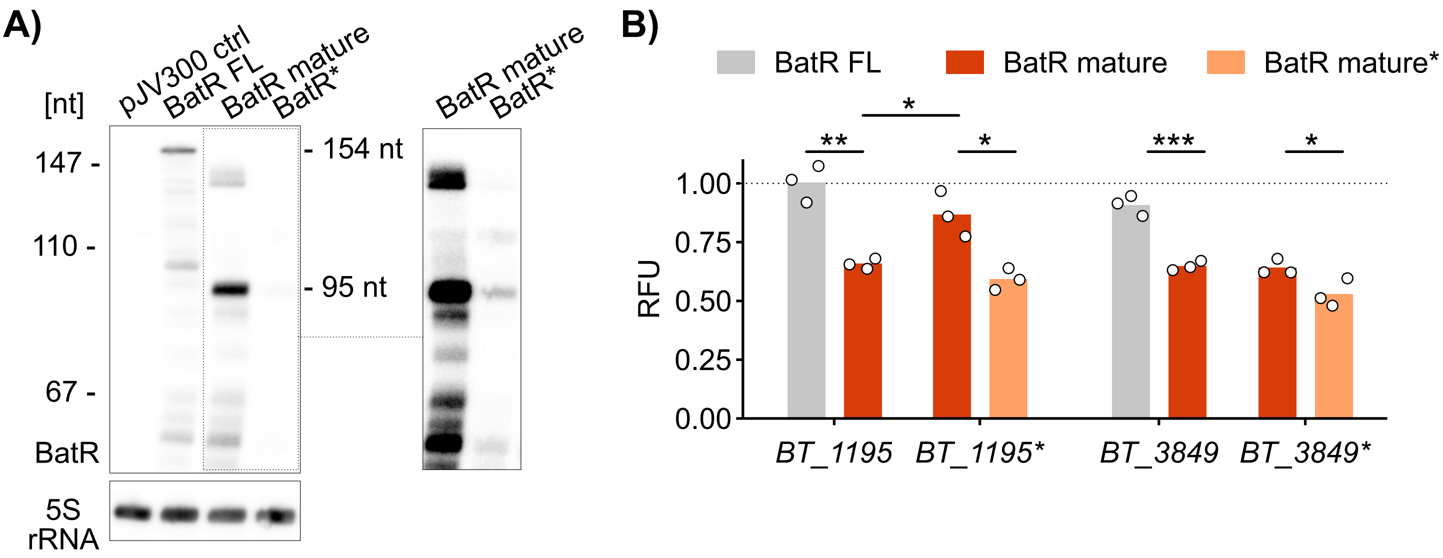


**Supplementary Figure S7**

A) Northern blot probing for BatR in E. coli strains containing the control plasmid (pJV300), or expressing the full-length (154 nt) or the mature (95 nt) BatR. The strain carrying mutated mature BatR (BatR*) carries a single G deletion in its promoter region that severely reduces transcription of this sRNA variant. An over-exposure of the same membrane is depicted on the right to visualize the signal derived from BatR*. FL: full-length.

B) Translational reporter assays with sfGFP fused to the predicted BatR interaction regions. Plotted is the GFP signal intensity normalized to the signal of the strain carrying the respective mRNA fusion and pJV300 plasmids and secondly, to the strain carrying the pXG30-sfGFP and respective pZE-BatR plasmids. An asterisk indicates a mutated BatR or target variant (see Supplementary Fig. S6A). See Fig. 6D for the control strains containing the original pXG30-sfGFP plasmid without gene fusions. Bars represent the average of three biological replicates, which are individually plotted as circles. ***: p-value < 0.001, **: p-value < 0.01, *: p-value < 0.05; unpaired t test.

## 3 MATERIAL AND METHODS

### *Bacterial strains and culture conditions*

Dataset S1 lists the strains, plasmids, oligonucleotides, and media used in this work. Bacteroides strains were grown at 37°C in an anaerobic chamber (Coy Laboratory Products) using an anaerobic gas mix of 85% N2, 10% CO2, and 5% H2. Solid media plates were made of supplemented BHI. Liquid cultures were grown in either rich (TYG) or minimal medium supplemented with 0.5% glucose (MM-G) (Dataset S1). For growth in rich media, a single colony of wild-type B. thetaiotaomicron VPI-5482 (AWS-001) was inoculated in 5 mL of TYG and incubated overnight (~ 16 h) at 37°C. For growth in MM-G, a single colony was inoculated in 5 mL and incubated for 24 h. Subcultures were made from these starter cultures in a 1:100 dilution into fresh medium.

### *PAM frequency determination*

A custom Python script was used for determining the abundance of selected PAMs among the intergenic sRNAs or the entire VPI-5482 genome sequence. The script counts the number of occurrences of each PAM on either strand of the input sequences. The code is available at https://github.com/gprezza/CRISPRi_tools.

### *Nuclease screening by in vitro transcription and translation (TXTL)*

Cleavage assays were performed as described previously (1). The plasmids encoding Cas12a, T7 RNA polymerase, and the CRISPR array were added into 9 µL of MyTXTL master mix (Arbor Biosciences, Cat # MYtxtl-70-96-M) to the same final concentration of 4.17 nM and a total volume of 12 µL. The mix was then incubated at 29°C for 16 h. Then, 1 µL of the mix, 1 µL of the targeted deGFP-encoding plasmid (concentration 10 nM), and 1 µL of nuclease-free water were added into 9 µL of MyTXTL master mix. Aliquots of 5 µL were placed in the wells of a 96-well V-bottom plate (Corning Costar, Cat # 3357) and incubated at 29°C for 16 h in a Synergy Neo2 microplate reader (BioTek, USA) with kinetic reading every 3 min (excitation 485 nm, emission 528 nm, gain 60, light source – Xenon Flash).

### *Western blotting*

Bacterial cultures grown in either TYG or MM-G media with the indicated IPTG concentration were collected at the indicated OD/time points, pelleted, and resuspended in Laemmli buffer at a concentration of 0.1 OD/mL. 20 µL of each sample were separated by 7% Tris Acetate PAGE and blotted on PVDF membranes. Equal loading was verified by staining the membrane with Ponceau S solution. FLAG-tagged protein signals were detected with anti-FLAG (Sigma-Aldrich, F1804) in combination with anti-mouse secondary antibody (Invitrogen, 31430).

### *Cloning of nucleases, gRNAs, and arrays*

All clonings were carried on in a Gibson assembly-like reaction with the NEBuilder® HiFi DNA Assembly kit (NEB, E2621) and into plasmids derived from pMM704 or pMM731 (2), a gift from Timothy Lu & Christopher Voigt (Addgene plasmids #68891, http://n2t.net/addgene:68891, RRID:Addgene_68891 and #68896, http://n2t.net/addgene:68896 , RRID:Addgene_68896). These vectors are based on the pNBU2 backbone, which integrates into the *Bacteroides* genome at either of the two *tRNA*$^{Ser}$ sites, ensuring preservation of a constant gene copy number of 1 across different transconjugants (3). The oligonucleotides used in the cloning reactions are indicated in Dataset S1.

Plasmids AWP-001 and AWP-002 were derived from pMM704 (without luciferase) and pMM731 (with luciferase), respectively, by replacing the Cas9 sequencce with an *Asc*I-*Not*I insertion region and the gRNA with a cassette formed by an FnCas12a repeat, a *Sma*I site, and the T1 terminator sequence of the *E. coli rnpB* gene. *E. coli* codon-optimized, FLAG-tagged Pb2Cas12a and dFnCas12a were PCR-amplified from plasmids pCB841 (internal number: 132282) (4) and pCB873 (1), respectively. dPb2Cas12a was generated by site-directed mutagenesis. The dPb2Cas12a or dFnCas12a cassette was inserted between the *Asc*I-*Not*I sites of plasmid AWP-002, resulting in the plasmids AWP-008 and AWP-006. AWP-029, the plasmid expressing dPb2Cas12a without the luciferase cassette, was cloned by inserting dPb2Cas12a into AWP-001 and is available from Addgene under ID 213966.

For cloning gRNAs, oligonucleotides comprising the 20 nt spacer sequence flanked 5′ by (5′-ATCTTTGCAGTAATTTCTACTGTTGTAGAT) and 3′ by (5′-CCGGCTTATCGGTCAGTTTCACCTGATTTA) homology arms were joined to *Sma*I-linearized AWP-008 or AWP-029 in a 10 µL reaction in a 20:1 oligonucleotide:vector ratio.

Three-spacer arrays were cloned through CRATES (1). Single-spacer arrays were constructed by inserting 30 nt spacers into either AWP-009 (*Francisella* repeats; 5′ arm: 5′- TTAAATAATTTCTACTGTTGTAGAT, 3′ arm: 5′-GTCTAAGAACTTTAAATAATTTGTC) or AWP-010 (*Prevotella* repeats; 5′ arm: 5′-TTATTTAATTTCTACTATTGTAGAT, 3′ arm: 5′- GGCTATAAAGCTTATTTAATTTCTA). The terminal repeat in *Prevotella* arrays is identical to the consensus repeat sequence (5), bearing the risk of generating "extraneous CRISPR RNAs" (ecrRNAs) (1) that would titrate Cas12a away from *bona fide* gRNAs and, possibly, result in off-targeting. As opposed to this, the terminal repeat in the *Francisella* array carries mutations that prevent ecrRNA generation (1).

All cloned plasmids were introduced in the donor *E. coli* S17-1 λpir strain. Transformants were conjugated with wild-type *B. thetaiotaomicron* and transconjugants counterselected on plates containing gentamicin and erythromycin.

***Luciferase assay***

Endpoint measurements of Nanoluc activity were performed as previously described (2) with minor modifications. An overnight culture of a Nanoluc- and dPb2Cas12a-expressing strain (containing a plasmid derived from AWP-008, AWP-009 or AWP-010) was subcultured 1:100 in fresh medium containing 250 µM IPTG to induce expression of the nuclease. Cultures were removed from the anaerobic chamber and put on ice at $OD_{600}$ 0.3-0.4 (~5 h). An aliquot of the culture was stored on ice for OD measurement at the end of the protocol. 500 µL of each sample were pelleted, washed once with PBS, and pelleted again, after which cells were lysed by resuspending in 50 µL of BugBuster reagent (Merck, 71456) and incubation under constant rotation for 10 min at room temperature. The lysate was cleared by centrifugation at 15,000 g for 15 min at 4°C and 45 µL of the supernatant were mixed with an equal volume of the NanoLuc Reagent (Promega N1110) prepared according to the manufacturer's instructions. After incubation for 5 min at 26°C, light emission of the sample was measured in 2 x 40 µL technical replicates on a Tecan infinite 200 PRO plate reader. Luciferase measurements were normalized to $OD_{600}$ values of 100 µL of the starting cultures, as measured in parallel on the infinite 200 PRO plate reader.

***RNA extraction, northern blotting, qRT-PCR***

Bacterial cultures were grown until the indicated OD and RNA was extracted as previously described (6). Briefly, 4 OD equivalents were collected in a tube, mixed with 20% volume of stop mix (7) (95%

ethanol, 5% water saturated phenol, pH >7.0) and snap-frozen in liquid nitrogen. After pelleting, cells were lysed with lysozyme (0.5 mg/mL) and 10% SDS in TE buffer. After addition of NaOAc (0.3 M final concentration), phenol (pH 4.5-5) was mixed at 1:1 volume and the samples were incubated for 6 min at 65°C, followed by addition of 1:1 volume of chloroform. After separation by centrifugation, nucleic acids in the aqueous phase were precipitated by adding 2 volumes of ethanol:3 M NaOAc (30:1 ratio) and incubating overnight at -80°C. After centrifugation, the pellet was resuspended in water and 40 µg of total RNA was cleared of contaminating DNA through treatment with 5 U of DNase I (Thermo Fisher Scientific #EN0521). RNA was again purified with a phenol-chloroform extraction followed by ethanol-NaOAc precipitation.

For northern blotting, 5 µg of total RNA was mixed with 1 volume of loading dye (95% formamide, 18 mM EDTA, 0.025% SDS, 0.025% xylene cyanol, 0.025% bromophenol blue) and denatured for 5 min at 95°C followed by 5 min incubation on ice. RNA was separated on a denaturing 6% polyacrylamide-7 M urea gel and electroblotted onto Hybond XL membranes (Amersham). Blotted membranes were probed with $^{32}$P-labeled gene-specific oligonucleotides (Dataset S1).

qRT-PCR was performed in a one-step reaction that includes reverse transcription with Takyon UF-NSMT-B0701 master mix and UF-RTAD-D0701 One-Step Kit Converter. The final volume of each reaction was 10 µL, containing 20 ng total RNA, 5 µL of qPCR mastermix, 0.1 µL of each primer (10 µM), and 0.1 µL of reverse transcriptase. Three technical replicates per sample were measured.

***Design and cloning of the CRISPRi library***

The script used to design the library is available at https://github.com/gprezza/CRISPRi_tools. Version v0.1.0 was used, with options *-a –nt*. A description of its criteria for selecting spacers and arrays can be found in the "Automated design of gRNAs against the full suite of intergenic *B. thetaiotaomicron* sRNAs" Results section. A Cas12a array is considered as properly folded if the probability of forming all three repeat hairpins is >20%, as predicted by RNAfold (8). The library was designed against a prior annotation of intergenic sRNAs, and some targeted sRNAs have since been reassigned to other categories (9). These sRNAs were filtered out during data analysis. The designed guide sequences are available in Dataset S2. The script provides oligonucleotides for cloning Cas12a three-spacer arrays with CRATES (1) reactions into AWP-031 (Addgene ID 213967), which was derived from AWP-029 by replacing the gRNA cassette with a GFP dropout system flanked by *Bsm*BI sites at both ends as in the original CRATES dropout construct. Restriction digestion leaves the overhangs CCTC and AACG at the 5′ and 3′ end, respectively. These overhang sequences are needed for CRATES and were taken from the "set 3" group of 25 unique four-base overhangs that were predicted to have > 95% fidelity in Gibson-like reactions in a previous study (10). The remaining 23 overhangs from the set allow cloning up to 23 arrays in a single CRATES reaction, in which each three-spacer array is generated from a set of six partially overlapping oligonucleotides (1). The final assembled array is flanked by the CCTC and AACG overhangs and contains internally one of the 23 overhangs, arranged so that only the full array comprising all three spacers can be formed. The script divided the set of 91 designed arrays in three sets of 23 arrays each (138 oligonucleotides) and a fourth set of 22 arrays (132 oligonucleotides).

These four pools were ordered as oPools™ oligo pools (IDT) and cloned into AWP-031 in four distinct CRATES reactions, with each containing all 138 or 132 oligonucleotides of the pool. The reactions were carried out similarly to the original protocol (1), with some modifications. Each oligonucleotide pool was phosphorylated at 37°C for 1 h, followed by a 65°C incubation for 20 min in a 40 µL reaction containing 5 µL of the pool (350 ng/µL, ~10 µM), 1.5 µL T4 Polynucleotide Kinase (Thermo #EK0031), 4 µL PNK buffer A, 4 µL of 10 mM ATP, and 25.5 µL of water. Phosphorylated oligonucleotide

pools were annealed by incubating for 5 min at 95°C, followed by cooling down to 85°C in steps of 30 s/degree, then to 65°C at 1 min/degree, and finally to 15°C at 30 s/degree. After this step, the nicks in the annealed fragments were repaired in a 50 µL reaction kept at 16°C for 5 h, followed by 65°C for 10 min and containing 40 µL of the annealed oligonucleotide pool, 2 µL of 10 mM ATP, 2.5 µL T4 DNA ligase (NEB #M0202), 0.55 µL PNK buffer A (Thermo), and 4.95 µL of water. We noticed that the assembled fragments comprised either the full-length three-spacer array or some shorter, misassembled sequences. We therefore gel-extracted the band corresponding to the size of the correctly assembled arrays (202 bp) and ligated these into already *Bsm*BI-digested AWP-031 in a reaction containing 80 ng digested AWP-031, 30 ng gel-extracted arrays, 1 µL T4 ligase buffer, 0.5 µL T4 DNA ligase, 0.5 µL *Bsm*BI, and water to 10 µL. The ligation was incubated in a thermocycler using the following program: 45 cycles of alternating digestion and ligation (42°C for 2 min, 16°C for 5 min) followed by a final digestion (55°C for 20 min) and an heat-inactivation step (80°C for 10 min).

Oligonucleotides for cloning gRNAs into AWP-029 are also provided by the script, comprising the 20 nt spacer sequence flanked by a 5′ (5′-ATCTTTGCAGTAATTTCTACTGTTGTAGAT) and a 3′ (5′-CCGGCTTATCGGTCAGTTTCACCTGATTTA) homology arm. The oligonucleotides containing the 557 spacers designed by the script were ordered as two oPools™ oligo pools (IDT) comprising 278 and 279 spacers each. The pools were cloned into *Sma*I-linearized AWP-029 with the NEBuilder® HiFi DNA Assembly kit (NEB, E2621) in two separate reactions as described above for the generation of single-gRNA constructs.

Ligated gRNA and array pools were separately transformed into the *E. coli* S17-1 λpir strain. We collected and pooled 1-2x$10^4$ colonies per transformation reaction and generated glycerol stocks. We inoculated each transformant pool in LB medium supplemented with ampicillin at a starting concentration of 0.04 OD, let grow until early exponential phase (~ OD 0.5), and mixed equal volumes of each array pool and each gRNA pool. We then mixed the array and gRNA *E. coli* cultures with wild-type *B. thetaiotaomicron* in early exponential phase (~ OD 0.7) at a 1:4:10 ratio, plated 10 OD of the mix on BHIS plates, and incubated aerobically overnight at 37°C. The following day, we resuspended the cell lawn in 1 mL of PBS and plated 100 µL aliquots of $10^{-1}$ and $10^{-2}$ dilutions on BHIS plates containing gentamicin (100 µg/mL) and erythromycin (12.5 µg/mL). We collected ~3x$10^5$ colonies and inoculated 70 mL of TYG supplemented with the same antibiotics at a starting OD of 0.08 OD. After the culture reached stationary phase (OD 4.2; ~24 h of incubation at 37°C), it was harvested and a glycerol stock was prepared. Three aliquots of the glycerol stock were processed for gDNA extraction and sequencing as described below. We considered library members with >10 read counts in all biological replicates as present in the final library.

### *CRISPRi screen under bile salt stress*

We spread an aliquot of the glycerol stock of the guide library on a BHIS plate and incubated for 2-3 d at 37°C. The day before the experiment, making sure to avoid single colonies, we harvested part of the cell lawn and used it to start an overnight culture in TYG medium at a starting OD of 0.05 OD/mL. We then diluted this input culture 1:100 into fresh TYG medium containing 250 µM of IPTG, with or without 0.5 mg/mL of bile salts (Fluka #48305). We incubated the subculture at 37°C until it reached the indicated OD, at which point we took a 2 mL aliquot, pelleted it, discarded the supernatant, and stored the bacterial samples at -20°C until further processing.

Bacterial pellets were resuspended in 100 µL of PBS containing 250 µg lysozyme, mixed with 100 µL lysis buffer (100 mM Tris-HCl pH 8.5, 200 mM NaCl, 0.2% SDS, 5 mM EDTA), and lysed by incubating at 37°C for 5 min. Proteins were then degraded by addition of 1 µL of Proteinase K (NEB

#P8107) and incubation at 56°C for 30 min, following which RNA was digested with 0.5 µL of RNase A (Thermo #EN0531) and incubation at 56°C for 5 min. The remaining gDNA was purified from each sample via phenol-chloroform extraction, followed by ethanol precipitation.

The genomic regions coding for gRNAs and arrays were PCR-amplified in a 50 µL reaction containing 100 ng gDNA with oligonucleotides AWO-577/AWO-578 (annealing temperature: 55°C) and AWO-575/AWO-576 (annealing temperature: 63°C).

***Sequencing and data analysis for the CRISPRi fitness screen***

Array and gRNA amplicons from the same gDNA sample were mixed at a 3:1 molar ratio and sequenced on an Illumina NextSeq 500 or NextSeq 2000 platform in paired-end mode, 150 bp reads. Data are available at the National Center for Biotechnology Information Gene Expression Omnibus database (https://www.ncbi.nlm.nih.gov/geo) under the accession number GSE235620. Sequenced reads were adapter- and quality- trimmed with BBDuk, using the following parameters: *k=13 mink=5 rcomp=t literal=ACACTCTTTCCCTACACGACGCTCTTCCGATCT,GTGACTGGAGTTCAGACGTGTGCTCTTCCGATCT hdist=1 ktrim=r minlen=100 tbo*. Trimmed reads were merged with BBMerge, using parameters *mininsert=120 maxloose=t*. The abundance of each library member within the merged reads was determined with a custom Python script (https://github.com/gprezza/CRISPRi_tools). Sequences of gRNAs and assembled arrays used for read mapping are available in Dataset S3.

Differential library member abundance analysis was performed with the R package edgeR (3.32.1), separately for gRNAs and arrays. In each case, guides with low abundance were filtered out with the filterByExpr function and undesired batch effects were removed with the RUVs function of the RUVSeq package (11). Normalization of the libraries was done with the *betweenLaneNormalization* function ("upper" method) of the EDASeq package. Fitness associated with the inhibition of each sRNA was calculated by averaging the fold-change of all constructs targeting the same sRNA, while their p-values were combined with Fisher's method. Differential library member abundance and sRNA fitness scores can be found in Dataset S4.

***Construction of batR deletion and complementation mutants and of BT_0521 or BT_1177 overexpression strains***

The *batR* gene was deleted from the *B. thetaiotaomicron* VPI-5482 genome as previously described (12). Briefly, we cloned ~750 bp of the regions upstream and downstream of the sRNA into the multiple cloning site of pSIE1 (a gift from Andrew Goodman, Addgene plasmid #136355; http://n2t.net/addgene:136355; RRID:Addgene_136355). The plasmid was then conjugated into *B. thetaiotaomicron*. Transconjugant colonies were grown over night in TYG medium and streaked on BHIS plates containing anhydrotetracycline. Mutants that had correctly excised the plasmid backbone were identified by colony PCR and the deletion and absence of unwanted mutations surrounding it were confirmed by Sanger sequencing. Complementation of BatR expression was achieved by first cloning the *batR* cassette, including its native promoter (the 50-nt region upstream of the transcription start site) and the sRNA sequence itself (the long, 374-nt isoform) including its intrinsic terminator between the *Xho*I and *Xba*I sites of the plasmid PWW542 (13), thereby replacing the GFP cassette. This generated plasmid AWP-089, which was conjugated into the Δ*batR* mutant background.

Overexpression of *BT_0521* or *BT_1177* was achieved similarly to the BatR complementation, with the exception that the strong phage promoter and ribosome binding site of PWW542 were kept

and only the CDSs were cloned downstream of them, replacing the GFP CDS. This gave rise to the plasmids AWP-090 (harboring *BT_0521*) and AWP-091 (*BT_1177*).

***B. thetaiotaomicron* growth curves**

Overnight cultures of a single colony of the indicated strains were diluted 1:100 into fresh TYG medium with or without 0.05 mg/mL of bile salts (Fluka #48305) and aliquoted in four 200 µL technical replicates in a transparent 96-well plate. Growth at 37°C was recorded by measuring absorbance (600 nm) in 20 min intervals with 10 s shaking before each measurement.

***sRNA homolog search, target prediction, and synteny analysis***

The sequence of BatR, including 50 nucleotides upstream of the sRNA's 5′ end to comprise the promoter, was searched with blastn (7 nt word size) (14) against the refseq_representative_genomes database filtered for Bacteroidetes (taxid 976). Sequences with <50% query cover were discarded and alignments were made with MAFFT (15). sRNA target prediction was done using the CopraRNA webserver (16) with default options, except for "nt up", which was set to 100 nt.

Synteny analysis was done on the region 5 kb up- and downstream of each BatR homologue (154 nt isoform). The annotated subgenomic regions were retrieved from the NCBI Nucleotide database and the synteny figure was generated with clinker (17).

***In-vitro transcription and 5′ end labelling***

RNAs were transcribed with the MEGAscript T7 kit (Ambion) according to the manufacturer's manual from templates prepared by PCR amplification of the respective genomic regions, with a forward primer containing the T7 promoter sequence. After transcription, samples were incubated with 1U TURBO DNase, Thermo Scientific for 15 min at 37°C. The RNA products were then separated on a 7 M urea 6% PAGE and bands of the correct size were cut out. RNA was eluted from the bands by overnight incubation at 4°C and 1,000 rpm shaking in 750 µL of RNA elution buffer (0.1 M NaAc, 0.1% SDS, 10 mM EDTA), following which the supernatant was subjected to phenol-chloroform extraction and ethanol precipitation to obtain the purified, *in vitro* transcribed RNAs. 50 pmol of each RNA were dephosphorylated by incubation for 1 h at 37°C with 25 U of calf intestine alkaline phosphatase (NEB) in a final volume of 50 µL, following purification by phenol-chloroform extraction and ethanol precipitation. 20 pmol of the obtained RNA were then radiolabeled by incubation with 1 U of Polynucleotide Kinase (NEB) and 20 µCi of $^{32}$P-γATP for 1 h at 37°C in a 20 µL reaction volume. Labeled RNA was purified on a G-50 column (GE Healthcare), following separation in and extraction from a polyacrylamide gel as described above.

***Electrophoretic mobility shift assay (EMSA)***

EMSAs were performed by incubating 4 nM of 5′ radiolabeled BatR with increasing concentrations (0, 3.9, 7.8, 15.6, 31.3, 62.5, 125, 250, 500 nM) of *in vitro*-transcribed mRNA fragments. Before incubation, the mRNAs were denatured by heating at 95°C for 1 min, followed by incubation on ice. The two RNA partners were then mixed in 1× structure buffer (10 mM Tris–HCl pH 7.0, 0.1 M KCl, 10 mM $MgCl_2$) containing 0.1 µg/µL of yeast RNA (Ambion) and incubated at 37°C for 1 h. Immediately prior loading, reactions were stopped by adding 3 µL of 5× loading dye (0.5× TBE, 50% glycerol, 0.2% xylene cyanol, 0.2% bromophenol blue). Samples were separated on a native 6% polyacrylamide gel, run at 4°C in 0.5% TBE buffer. After 3 h the run was stopped, gels were dried, and exposed on phosphor screens.

The dissociation constant ($K_d$) was calculated by fitting the values of the fraction of bound sRNAs ($F_{bound}$) to an exponential curve of formula $F_{bound} = B_{max} * mRNA_{conc} / (K_d + mRNA_{conc})$, where $B_{max}$ is the maximum specific binding and $mRNA_{conc}$ the concentration of mRNA fragment.

***GFP reporter assay in E. coli TOP10***

Translational fusion reporters were constructed as described previously (18, 19). All four BatR target candidates are part of operons, with the respective target protein being encoded from an internal CDS of the cognate polycistronic mRNA. Therefore, fragments comprising the C-terminus of the upstream ORF, the intergenic region, and the region coding the N-terminus of the protein of interest were PCR-amplified and cloned into the *Nsi*I-*Nhe*I sites of the pXG30-SF plasmid (19), generating a translational fusion of the gene of interest with superfolder GFP. Expression plasmids of the long (full-length, 154-nt) and mature (95-nt isoform) BatR sRNA variants were generated as follows. The vector backbone of plasmid pZE12-luc (20) was PCR amplified with oligonucleotides pLlacoB and pLlacoD, and digested with *Xba*I. The resulting ~2.2 kb-long fragment was gel-extracted and ligated to the sRNA fragments, which were generated by PCR with primers containing a 5′ phosphate (forward) and an *Xba*I site (reverse). Nucleotide exchanges in the GFP fusion constructs and sRNA expression plasmids were generated by overlapping PCR. The resulting plasmids were cotransformed into *E. coli* TOP 10 electrocompetent cells. All oligonucleotides used for PCRs and the generated strains are listed in Dataset S1.

Single colonies of each strain were grown over night in LB medium at 37°C. On the next day, 0.2 ODs of each culture were collected and pelleted. After removal of the supernatant, the pellet was resuspended in 150 µL of 4% paraformaldehyde and stored at room temperature in the dark for 30 min. Cells were washed once in 200 µL of PBS and resuspended in the same volume of PBS. A 1:100 dilution of this final suspension was subjected to flow cytometry (Agilent NovoCyte Quanteon). After excluding events with a FSC-H < 2,000 (salt particles and cellular debris), GFP median intensity was recorded over 50,000 events per strain.

## 4 LEGENDS FOR DATASETS S1 to S6

**Dataset S1**

Oligonucleotides, plasmids, strains, and media used in this study.

**Dataset S2**

Oligonucleotides used for cloning the CRISPRi library.

**Dataset S3**

Assembled sequences of all gRNAs and arrays in the CRISPRi library in FASTA format.

**Dataset S4**

CRISPRi fold change data upon bile stress per each library member (first sheet) or sRNA (second sheet).

**Dataset S5**

CopraRNA target predictions for BatR.

**Dataset S6**

CopraRNA target predictions for BTnc353.

## 5 SI REFERENCES


1. C. Liao *et al.*, Modular one-pot assembly of CRISPR arrays enables library generation and reveals factors influencing crRNA biogenesis. *Nature communications* **10**, 2948 (2019).
2. M. Mimee, A. C. Tucker, C. A. Voigt, T. K. Lu, Programming a Human Commensal Bacterium, Bacteroides thetaiotaomicron, to Sense and Respond to Stimuli in the Murine Gut Microbiota. *Cell Syst* **1**, 62-71 (2015).
3. J. Wang, N. B. Shoemaker, G. R. Wang, A. A. Salyers, Characterization of a Bacteroides mobilizable transposon, NBU2, which carries a functional lincomycin resistance gene. *J Bacteriol* **182**, 3559-3571 (2000).
4. R. Marshall *et al.*, Rapid and Scalable Characterization of CRISPR Technologies Using an E. coli Cell-Free Transcription-Translation System. *Mol Cell* **69**, 146-157 e143 (2018).
5. C. Pourcel *et al.*, CRISPRCasdb a successor of CRISPRdb containing CRISPR arrays and cas genes from complete genome sequences, and tools to download and query lists of repeats and spacers. *Nucleic Acids Res* **48**, D535-D544 (2020).
6. D. Ryan, L. Jenniches, S. Reichardt, L. Barquist, A. J. Westermann, A high-resolution transcriptome map identifies small RNA regulation of metabolism in the gut microbe Bacteroides thetaiotaomicron. *Nature communications* **11**, 3557 (2020).
7. K. Tedin, U. Blasi, The RNA chain elongation rate of the lambda late mRNA is unaffected by high levels of ppGpp in the absence of amino acid starvation. *J Biol Chem* **271**, 17675-17686 (1996).
8. R. Lorenz *et al.*, ViennaRNA Package 2.0. *Algorithms Mol Biol* **6**, 26 (2011).
9. D. Ryan *et al.*, An integrated transcriptomics-functional genomics approach reveals a small RNA that modulates Bacteroides thetaiotaomicron sensitivity to tetracyclines. *bioRxiv* 10.1101/2023.02.16.528795 (2023).
10. V. Potapov *et al.*, Comprehensive Profiling of Four Base Overhang Ligation Fidelity by T4 DNA Ligase and Application to DNA Assembly. *ACS Synth Biol* **7**, 2665-2674 (2018).
11. D. Risso, J. Ngai, T. P. Speed, S. Dudoit, Normalization of RNA-seq data using factor analysis of control genes or samples. *Nat Biotechnol* **32**, 896-902 (2014).
12. N. A. Bencivenga-Barry, B. Lim, C. M. Herrera, M. S. Trent, A. L. Goodman, Genetic Manipulation of Wild Human Gut Bacteroides. *J Bacteriol* **202** (2020).
13. W. R. Whitaker, E. S. Shepherd, J. L. Sonnenburg, Tunable Expression Tools Enable Single-Cell Strain Distinction in the Gut Microbiome. *Cell* **169**, 538-546 e512 (2017).
14. S. F. Altschul *et al.*, Gapped BLAST and PSI-BLAST: a new generation of protein database search programs. *Nucleic Acids Res* **25**, 3389-3402 (1997).
15. K. Katoh, D. M. Standley, MAFFT multiple sequence alignment software version 7: improvements in performance and usability. *Mol Biol Evol* **30**, 772-780 (2013).
16. P. R. Wright *et al.*, CopraRNA and IntaRNA: predicting small RNA targets, networks and interaction domains. *Nucleic Acids Res* **42**, W119-123 (2014).
17. C. L. M. Gilchrist, Y. H. Chooi, clinker & clustermap.js: automatic generation of gene cluster comparison figures. *Bioinformatics* **37**, 2473-2475 (2021).
18. J. H. Urban, J. Vogel, Translational control and target recognition by Escherichia coli small RNAs in vivo. *Nucleic Acids Res* **35**, 1018-1037 (2007).
19. C. P. Corcoran *et al.*, Superfolder GFP reporters validate diverse new mRNA targets of the classic porin regulator, MicF RNA. *Molecular microbiology* **84**, 428-445 (2012).
20. R. Lutz, H. Bujard, Independent and tight regulation of transcriptional units in Escherichia coli via the LacR/O, the TetR/O and AraC/I1-I2 regulatory elements. *Nucleic Acids Res* **25**, 1203-1210 (1997).
